# Spatiotemporal, optogenetic control of gene expression in organoids

**DOI:** 10.1038/s41592-023-01986-w

**Published:** 2023-09-21

**Authors:** Ivano Legnini, Lisa Emmenegger, Alessandra Zappulo, Agnieszka Rybak-Wolf, Ricardo Wurmus, Anna Oliveras Martinez, Cledi Cerda Jara, Anastasiya Boltengagen, Talé Hessler, Guido Mastrobuoni, Stefan Kempa, Robert Zinzen, Andrew Woehler, Nikolaus Rajewsky

**Affiliations:** 1 Laboratory for Systems Biology of Gene Regulatory Elements, Berlin Institute for Medical Systems Biology (BIMSB), Max Delbrück Center for Molecular Medicine (MDC) in the Helmholtz Association, Berlin, Germany; 2 Systems Biology of Neural Tissue Differentiation, Berlin Institute for Medical Systems Biology (BIMSB), Max Delbrück Center for Molecular Medicine (MDC) in the Helmholtz Association, Berlin, Germany; 3 Organoid Platform, Berlin Institute for Medical Systems Biology (BIMSB), Max Delbrück Center for Molecular Medicine (MDC) in the Helmholtz Association, Berlin, Germany; 4 Bioinformatics and Omics Data Science, Berlin Institute for Medical Systems Biology (BIMSB), Max Delbrück Center for Molecular Medicine (MDC) in the Helmholtz Association, Berlin, Germany; 5 Systems Biology Imaging Platform, Berlin Institute for Medical Systems Biology (BIMSB), Max Delbrück Center for Molecular Medicine (MDC) in the Helmholtz Association, Berlin, Germany; 6 Proteomic and Metabolomics Platform, Berlin Institute for Medical Systems Biology (BIMSB), Max Delbrück Center for Molecular Medicine (MDC) in the Helmholtz Association, Berlin, Germany; 7 Howard Hughes Medical Institute, Janelia Research Campus, Ashburn, VA USA; 8 Charité–Universitätsmedizin, Berlin, Germany; 9 German Center for Cardiovascular Research (DZHK), Berlin, Germany; 10 NeuroCure Cluster of Excellence, Berlin, Germany; 11 National Center for Tumor Diseases (NCT), German Cancer Consortium (DKTK), Berlin, Germany; 12 Present Address: Human Technopole, Milan, Italy

**Keywords:** Optogenetics, Pattern formation, Transcriptomics, Stem cells

## Abstract

Organoids derived from stem cells have become an increasingly important tool for studying human development and modeling disease. However, methods are still needed to control and study spatiotemporal patterns of gene expression in organoids. Here we combined optogenetics and gene perturbation technologies to activate or knock-down RNA of target genes in programmable spatiotemporal patterns. To illustrate the usefulness of our approach, we locally activated Sonic Hedgehog (*SHH*) signaling in an organoid model for human neurodevelopment. Spatial and single-cell transcriptomic analyses showed that this local induction was sufficient to generate stereotypically patterned organoids and revealed new insights into *SHH*’s contribution to gene regulation in neurodevelopment. With this study, we propose optogenetic perturbations in combination with spatial transcriptomics as a powerful technology to reprogram and study cell fates and tissue patterning in organoids.

## Abstract (web summary)

A workflow combining optogenetic perturbations with spatial transcriptomics to program spatiotemporal gene expression patterns in organoids.

## Main

Organoid culture has proved to be a transformative technology by offering the opportunity to access unique features of human development and to model complex attributes of human disease in vitro. Organoid development relies on the intrinsic property of stem cells to differentiate and self-organize in three-dimensional (3D) space^1^. Specific developmental trajectories can be promoted by treatment with signaling molecules or by genetic manipulations, and more complex architectures can be achieved by fusing different organoids into assembloids^2^. Controlling organoid patterning and gene expression in a spatiotemporally programmable manner, however, remains challenging.

Methods such as single-cell RNA sequencing (scRNA-seq) and spatial transcriptomics have proved immensely useful to describe the molecular signatures of cell states and their relationships within tissues. However, to explain the molecular mechanisms that explain these data, it is essential to conditionally perturb gene expression, at single-cell resolution and in both time and space.

To address the need for spatiotemporally programmable gene perturbations, we developed a flexible system that allows light-inducible activation and repression of target genes, by combining optogenetic transcription^3–5^ with CRISPR–Cas13 knock-downs^6–8^. We then engineered live-cell photostimulation setups to ‘print’ complex patterns of gene expression onto cultured cells and organoids.

To show that our approach can control organoid patterning, we chose to locally activate the Sonic Hedgehog (SHH) pathway in an organoid model of neurodevelopment, and quantified the impact of this perturbation with single-cell and spatial transcriptomics. SHH is a well-studied morphogen, essential in a variety of biological processes. In the developing vertebrate neural tube, it specifies distinct cellular fates along the dorsoventral (DV) axis^9^. We generated neural organoids where we locally induced *SHH* in a spatially determined organizer.

Spatial transcriptomics revealed that this was sufficient to establish distinct gene expression territories, resembling those found in the ventral regions of the neural tube in vivo. Single-cell analysis allowed us to more comprehensively reconstruct DV identities corresponding to the neural tube patterning and revealed potentially interesting insights into SHH activity, for example, the induction of IGF pathway modulators, the differentiation of cells expressing pericyte markers and the spatial modulation of axon guidance genes.

## Results

### Light-inducible gene activation and knock-down

To perturb RNA expression with spatial resolution, we adopted, constructed and optimized a variety of tools based on the combination of light-inducible proteins to allow spatial control by photostimulation, with gene perturbation effectors to induce gene activations and knock-downs. Such design consists of an activation module, based on light-inducible CRISPR–Cas9, TetON or Cre–Lox systems, which can be used to activate endogenous promoters or exogenous expression cassettes, and a CRISPR–Cas13 module, coupled with the activation module for knocking down transcripts of interest.

**Fig. 1:**
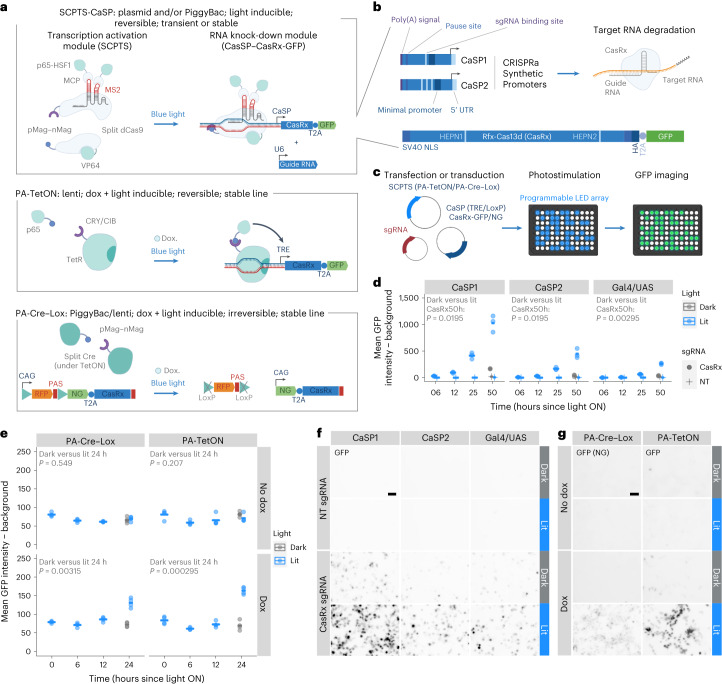
Light-inducible gene activation and knock-down modules. **a**, The top shows the light-inducible transcription activation module (SCPTS), based on a dCas9 fused to transcription activation domains, driving CasRx transcription in this case. A U6 promoter-driven CasRx guide RNA can be co-expressed. The middle shows PA-TetON system. The bottom shows PA-Cre–Lox. **b**, Synthetic promoters for light-inducible transcription of CasRx or any other cassette of interest (CaSP1/2), containing upstream elements for reducing spurious transcription (poly(A) site, pause site), a minimal CMV promoter containing TFIIB binding site/TATA box, an initiator and synthetic 5′ untranslated region (UTR), one or three sgRNA binding sites. The CasRx cassette (below) contains a T2A-GFP tag, two nuclear localization signals (NLS) and an HA tag, as in ref. ^8^. Catalytic domains (HEPN) are indicated. **c**, Experimental setup: HEK293T cells are transfected in a 96-well plate, which is placed on a LED board for photostimulation and cells are then imaged for GFP/NeonGreen (NG). **d**, For the SCPTS system, background-subtracted mean GFP intensity at indicated time points (dark or lit), with one of the three promoters (CaSP1/2, Gal4/UAS), either a nontargeting (NT) or the CaSP–CasRx-targeting guide (CasRx). Horizontal bars show the mean of all replicates per condition; dots show individual replicates. *P* values (Benjamini–Hochberg corrected two-sided *t*-test) between dark and lit conditions at 50 h are reported. **e**, Same as **d**, for the PA-TetOn and PA-Cre–Lox systems, with or without doxycycline (*n* = 4). *P* value (Benjamini–Hochberg corrected two-sided *t*-test) between dark and lit conditions at 24 h are reported. **f**,**g**, Representative images for the SCPTS (GFP) (**f**), PA-Cre–Lox (NG) and PA-TetON (GFP) (**g**). Scale bars, 50 μm. Images were taken at 36 h posttransfection using a Keyence BZ-X710 with ×10 magnification, in *n* = 6 independent experiments from those in **d**,**e**. Brightfield in Extended Data Fig. 1k. Source data

**Extended Data Fig. 1:**
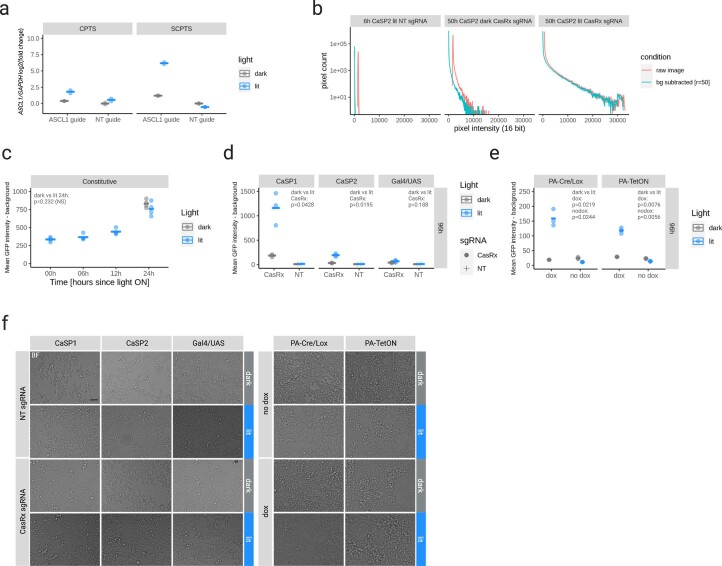
Light-inducible gene activation and knock-down modules. **a**. Log2 fold-change of the *ASCL1* mRNA, induced with the CPTS (left) and SCPTS (right) systems^3^, between a non-targeting (NT) and promoter-targeting sgRNA (*ASCL1*), in dark or lit conditions at 24h, measured by RT-qPCR. Each dot represents a biological replicate (n = 2). **b**. Histograms of pixel intensity with and without background correction (green and red), performed with the subtract background function in Fiji/imageJ with a rolling ball radius of 50, for three representative samples of the SCPTS CaSP2 CasRx system (left: 6h time point with non-targeting guide, middle: 50h time point with the Tet6 targeting guide in the dark, right: 50h time point with the Tet6 targeting guide with illumination). **c**. Background-subtracted mean GFP intensity, in either dark or lit conditions (n = 4) for constitutive CasRx. P-values (Benjamini-Hochberg corrected two-sided t test) between dark and lit conditions at 50h are shown. **d-e**. As in Fig. 1d, e, for an additional time point at 96h (n = 3, except for NT guide control n = 1). Each dot represents a replicate (n = 3, except for NT guide n = 1) and horizontal lines represent means over each condition. P-values (Benjamini-Hochberg corrected two-sided t test) between dark and lit condition for CasRx guide and dox/no dox conditions are shown. **f**. Brighfield images corresponding to the panels in Fig. 1f, g. Scale bar (top left panel, applies to all): 50 μm. Horizontal lines represent the mean of all replicates in each plot. Source data

To optogenetically activate genes, we first used the split CRISPR–Cas9-based photoactivatable transcription system (SCPTS^3^), which consists of an enzymatically dead Cas9 (dCas9) split into N- and C-terminal domains and fused to the photoinducible dimerization moieties pMag and nMag. Blue light triggers pMag–nMag dimerization, thereby reconstituting dCas9, that uses guide RNAs to bind a given promoter. The SCPTS system activates transcription nearby (CRISPR activation, CRISPRa), via fused or associating domains (VP64 and p65-HSF1, fused to MCP and tethered on the single-guide RNA (sgRNA) via MS2 motifs; Fig. 1a, top panel). This system has been shown to be a potent transcriptional activator under blue light illumination, for example, by inducing the expression of the neuronal transcription factor ASCL1 (ref. ^3^) (reproduced here; Extended Data Fig. 1a). We established the SCPTS system in human embryonic kidney (HEK) cells, transfecting the plasmids encoding the systems’ components and using a programmable light-emitting diode (LED) board for photostimulation. This setup consists of an array of 96 blue LEDs mounted in a scaffold that can be placed in a cell culture incubator and accommodate a 96-well cell culture plate. LEDs can be programmed individually via a microcontroller (Methods).

We tested a promoter–sgRNA pair, previously used in a similar context (Gal4/UAS (ref. ^10^)), and additionally designed two synthetic promoters (CRISPRa Synthetic Promoter: CaSP1 and 2, partially based on ref. ^11^; Fig. 1b) to drive transcription of any given expression cassette. In this case, we activated the expression of a green fluorescent protein- (GFP-)tagged CasRx, which can be programmed for RNA knock-downs. We transfected HEK cells with the plasmids encoding these components and imaged GFP over time on photostimulation (Fig. 1c and Extended Data Fig. 1b). Both synthetic promoters were more active than the UAS. CaSP1 induced a roughly 45-fold-change activity over a nontargeting guide control after 50 hours of photostimulation, with roughly 16% leakage in the dark, while CaSP2 elicited a roughly 21-fold induction with around 9% leakage (Fig. 1d). A constitutive promoter produced GFP with no substantial difference on illumination (Extended Data Fig. 1c).

We also used a light-inducible TetON system, consisting of a TetR/p65 transactivator fused with CRY/CIB photodimers^4^, and Cre–Lox^5^, based on a split Cre fused with pMag–nMag photodimers. The PA-TetON system is coupled with a Tet-Responsive Element (TRE) controlling CasRx-GFP, the PA-Cre–Lox system is combined with a LoxP-RFP–LoxP-NeonGreen-CasRx cassette, which places NeonGreen-CasRx under a CAG promoter upon the Cre-mediated removal of the red fluorescent protein (RFP) cassette and its poly(A) site, and they both require a double switch (light and doxycycline). For the first, we cloned a CasRx–T2A-GFP cassette under a TRE promoter and transduced HEK cells with two lentiviruses expressing these components, while for the Cre–Lox system we used two PiggyBac vectors, one carrying the split Cre and the other a LoxP-RFP-LoxP cassette followed by NeonGreen-T2A-CasRx (Fig. 1a, middle and lower panels). Both systems proved reasonably tight and light-responsive, with significant activation over the dark and no-doxycycline controls (Fig. 1e). Representative microscopy images, additional time points and flow cytometry validations are shown in Fig. 1f–g, Extended Data Fig. 1d–f and Supplementary Fig. 1.

An extensive report of the knock-down module efficacy and additional attempts at designing light-inducible Cas13 proteins is reported in Supplementary Note and in Supplementary Figs. 2 and 3.

### Spatial programming of optogenetic stimulations

**Fig. 2:**
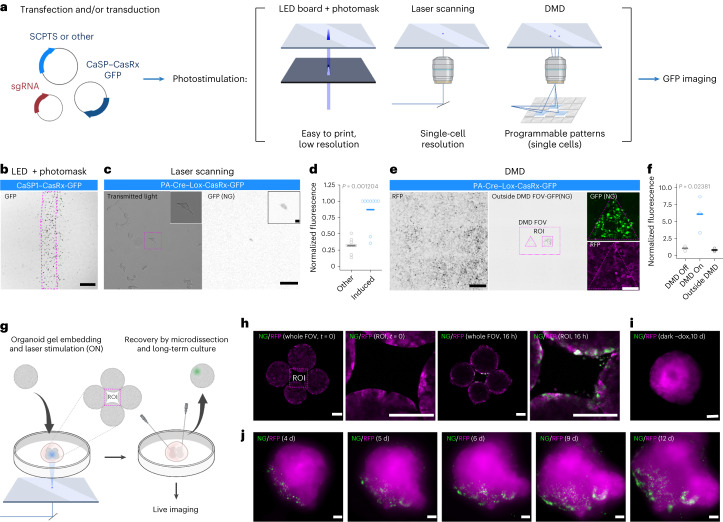
Spatial programming of optogenetic stimulations. **a**, Spatial photostimulations: cells are stimulated with a LED array through a photomask, with laser scanning or with a DMD. **b**, Representative images (*n* = 3) of LED stimulation of the CaSP1–CasRx system in HEK cells (NG). Magenta, photomask. Scale bar, 500 μm. **c**, Single-cell Cre–Lox CasRx laser stimulation in HEK cells. The left shows the transmitted light. The right shows the NG. Scale bars, 100 μm. Top right shows a higher magnification. Magenta, photostimulated ROI. **d**, NG quantification for an ROI covering the photostimulated cell (blue, induced) and for the mean of ROI of the same size tiling the entire FOV 16–18 h after stimulation (gray, other). Each dot is a replicate (*n* = 9 induced cells in five different experiments), horizontal bars represent the mean and *P* value for induced versus other comparison is shown (two-sided Wilcoxon–Mann–Whitney). **e**, Representative image of a complex pattern stimulation of the Cre–Lox CasRx system in HEK cells performed with the DMD setup. The left shows the RFP, the middle shows the NG and the right shows a zoom-in of an individual ROI. Photostimulated ROI and entire DMD FOV (rectangle outside) in magenta. The broader FOV (outside DMD) was imaged 24 h post-photostimulation with a confocal setup. Scale bars, 500 μm. **f**, Quantification of NG for the DMD ROI (DMD On), the DMD FOV outside the photostimulated ROI (DMD Off) and the imaged region outside the DMD FOV (Outside DMD). Each dot is a replicate (*n* = 3), horizontal bars represent the mean and *P* value for the DMD On versus both DMD Off and Outside DMD is shown (two-sided Wilcoxon–Mann–Whitney). **g**, Organoid photostimulation protocol: four organoids are placed on a glass-bottom dish, immobilized with a Geltrex droplet and photostimulated with laser scanning overnight. **h**, From left to right: representative (*n* = 7) live imaging (whole FOV and photostimulated ROI) of four organoids at time 0 and after 16 h of pulsed photostimulation. Magenta, RFP; green, NG. Scale bars, 100 μm. **i**, Live imaging of a representative control organoid (dark) at 10 days. Scale bar, 250 μm. Magenta, RFP; green, NG. **j**, Live imaging of a representative time course (4–12 days, *n* = 3) of an organoid locally photostimulated via laser scanning. Magenta, RFP; green, NG. Scale bars, 100 μm. Source data

To leverage the potential of optogenetic RNA perturbations, we not only need programmable gene perturbation modules, but also the means to program them spatially. To this end, we tested three different approaches (Fig. 2a): photomasks, directed laser stimulations and a programmable digital micromirror device (DMD).

**Extended Data Fig. 2:**
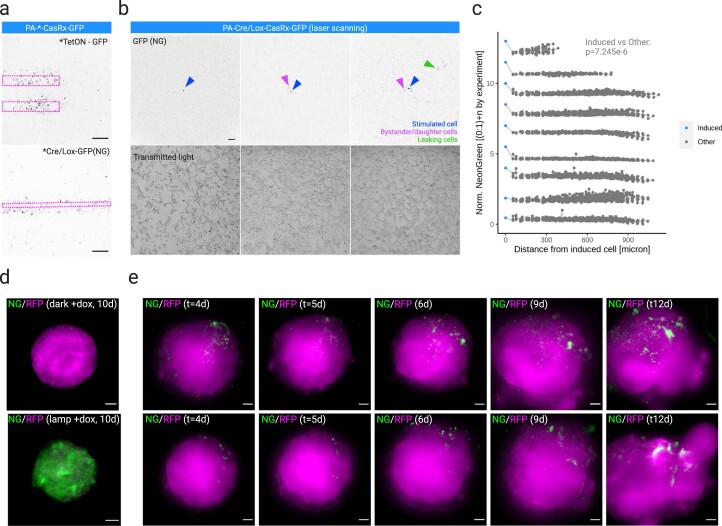
Spatial programming of gene expression. **a**. As in Fig. 2b, representative (n = 3) fluorescent microscope image of a photomask stimulation for the PA-TetON (top) and PA-Cre/Lox (bottom) CasRx systems in HEK cells. Magenta: photomask shape. Signal is NG in gray scale. Scale bar: 500 μm. **b**. As in Fig. 2c, representative (n = 9 single cells from 5 different experiments) images of a single-cell Cre/Lox CasRx stimulation in HEK cells, performed with 100 Hz laser scanning within a confocal microscope setup. Top: confocal image of GFP in grey scale, bottom: transmitted light image. Scale bar: 100 μm. The three FOVs are larger than in Fig. 2c and represent three typical scenarios observed in single-cell photo-stimulations. Left: a single cell is successfully induced; middle: bystander cells are induced as well (moving through the illuminated FOV or deriving by cell division); right: additional cells elsewhere in the FOV are also induced (‘leakage’). **c**. NG fluorescence quantification for all replicates of single-cell stimulations. A grid of ROIs of ca. the size of a single cell spanning the entire FOV is used for quantifying the signal of the photo-stimulated cell (left ROI, in blue), and of all other ROIs in the FOV (grey), sorted by their distance in micrometers from the photo-stimulated ROI. Signal is normalized (0 to 1) and each replicate is summed with an arbitrary integer to show them stacked (n = 9 FOVs photo-stimulated in 5 experiments). P-value: two-sided t-test. **d**. Representative (n = 3) live imaging of an organoid treated with 1 μg/ml doxycycline for 24 h and kept in the dark (top) or photo-stimulated with a LED lamp for 24 h (bottom), at 10 days post-stimulation. Magenta: RFP; Green: NeonGreen. Scale bar: 250 μm. **e**. Live imaging of a time-course as in Fig. 2j for two more organoids locally photo-stimulated via laser scanning (n = 3). Magenta: RFP; Green: NeonGreen. Scale bar: 100 μm.

First, we applied LED board stimulations to HEK cells transfected or transduced with all three systems (SCPTS, TetOn and Cre–Lox). The array is capable of generating patterns of activation with a resolution on the order of hundreds of micrometers, provided by a photomask placed between the LEDs and the plate (Fig. 2b and Extended Data Fig. 2a). However, in addition to the previously observed leakage, we noticed some diffused induction beyond the edges of the mask, possibly due to reflection and refraction within the cell culture dish. This approach, while simple to construct and intuitive, provides a narrow application range and is largely limited to thin two-dimensional (2D) cultures.

We therefore tested whether we could achieve precise spatial activation of gene expression at higher resolution with a laser scanning-based photostimulation approach. We induced CasRx-GFP expression with the Cre–Lox system by stimulating a region of interest (ROI) containing a single cell. Using this approach, we could stimulate a single cell in a field of view (FOV) containing several cells (Fig. 2c). This approach presented also some limitations: in a few cases, the photostimulated cell was not induced within the 16–18 hour-long program that we used (for example, by moving away from the illuminated ROI), while in other cases we observed some cell division during the photostimulation, as well as some bystander cells transiently moving through the ROI, and few activated cells elsewhere in the FOV due to leakage (Fig. 2d and Extended Data Fig. 2b,c).

Additionally, we constructed a DMD microscope, combined with a cell culture chamber for live-cell stimulation and imaging. The DMD is controlled by a simple MicroManager and ImageJ-based graphical user interface, which allows to intuitively program spatial activation patterns. With this setup, we can draw multiple ROI with different shapes, which will then be illuminated in parallel with the desired spatiotemporal patterns. Photostimulation in the designed ROI elicits robust activation of the light-inducible gene expression cassette as compared to the rest of the DMD FOV, as well as to cells located outside it (Fig. 2e,f).

Last, we optimized a protocol for spatially restricted photostimulations in organoids (Fig. 2g). We used the laser scanning setup and achieved robust activation with a 10–16 hour pulsed photostimulation pattern. To hold organoids firm during the stimulation, we embedded them in a gel droplet onto a glass-bottom dish and retrieved them by microdissection after the stimulation (Fig. 2g). Imaging before and after stimulation (Fig. 2h) shows robust and specific activation of the system in the illuminated region, while no activation is observed even after several days in control organoids (Fig. 2i and Extended Data Fig. 2d). Live imaging up to several days poststimulation shows that the activated cells proliferate and migrate, but the nuclear NeonGreen signal remains overall polarized (Fig. 2j and Extended Data Fig. 2e).

### Optogenetic stimulation of the SHH pathway in hiPSCs

**Extended Data Fig. 3:**
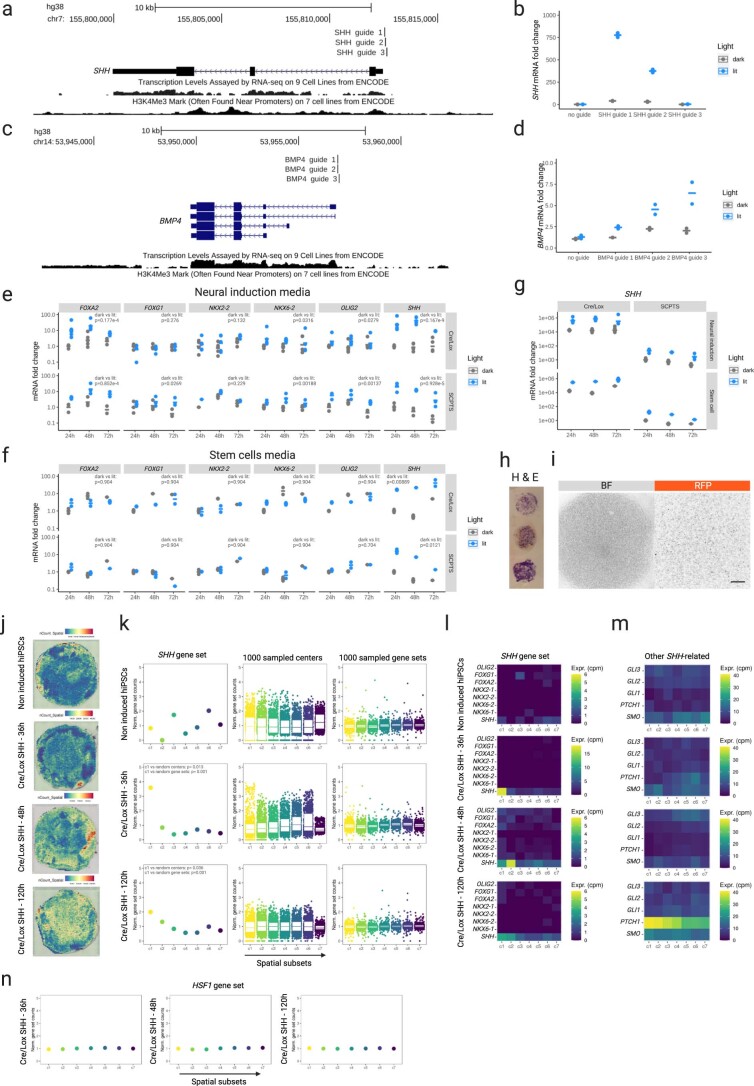
Optogenetic stimulation of Sonic Hedgehog in human stem cells. **a**. UCSC genome browser window of human *SHH* locus with transcription and H3K4Me3 tracks from ENCODE and three sgRNAs used for SCPTS. **b**. *SHH* mRNA (RT-qPCR) in HEK293T cells transfected with the SCPTS system without or with one of the three *SHH* promoter-targeting sgRNAs, dark vs 24h lit. Values are fold change over no-guide dark control, normalized on *GAPDH*. Dots represent each biological replicate (n = 2) and horizontal bars the mean of all replicates. **c, d**. As a-b, for *BMP4*. **e**. RT-qPCR measurement of the mRNAs indicated on top in hiPSCs induced for *SHH* expression with the SCPTS loaded with the *SHH* guide 1 or the Cre/Lox system, with or without light stimulation, for 24–72h. Data represent fold change over 24h dark control. Dots represent each biological replicate (n = 2–5) and horizontal bars the mean over each condition. Benjamini-Hochberg corrected two-sided t test p-values are computed between dark and lit. **f**. As in e, but with stem cell media instead of neural induction media. **g**. *SHH* mRNA (RT-qPCR) using the SCPTS system and *SHH* guide 1 (right) or the Cre/Lox system (left), dark vs lit, for 24–72h. Data are the same as in e-f, but plotted as fold-change over the SCPTS dark 24h control for both series (SCPTS and Cre/Lox), to compare the two. **h**. Representative H&E staining on n = 3 replicates of hiPSCs cultured on a PET membrane and transferred onto a glass slide. **i**. Brightfield (left) and RFP (right) channels for hiPSCs Cre/Lox *SHH* cultured as a monolayer on a PET membrane, induced in the center with a circular photomask (Fig. 3c, n = 1 per time point). Scale bar: 500 μm. **j**. UMI counts for the Visium capture areas in the 4 hiPSC Cre/Lox SHH samples at 0,36,48 and 120h of induction. **k**. Left: normalized counts of a *SHH* gene set, obtained by adding the counts of each gene in the set in each of the 7 concentric circles (c1-7), normalized by the total transcript counts per circle. Middle left: same sampling 1000 times a random central spot. Middle right: same sampling 1000 times a random gene set on the same concentric circles of the left panel. Right: heatmap showing transcript counts per million (cpm) for all genes in the *SHH* gene set across the 7 concentric circles. Data shown here for hiPSCs Cre/Lox *SHH* induced for 0, 36 and 120h, while 48h is shown in Fig. 4e (n = 1 per time point for 4 time points). Exact p-values < 0.05 are indicated, computed as the fraction of values exceeding the tested value from random sampling of 1000 centers and 1000 gene sets. **l**. Heatmap showing transcript counts per million (cpm) for the individual genes in the the *SHH* pathway across the 7 concentric circles (c1-7) and the 4 samples (induction of 0h, 36h, 48h and 120h). **m**. Same as k, for additional genes involved in the SHH pathway. **n**. Same as k, for a gene set comprised of target genes of the heat shock transcription factor HSF1. All boxplots: center and bounds represent median, 25% and 75% quantiles. Source data

To test the ability of our setup to perturb biologically relevant processes, we focused on the induction of SHH signaling in stem cells and organoids. We first designed three sgRNAs for activating the endogenous *SHH* promoter with the SCPTS system (Extended Data Fig. 3a). We transfected HEK cells with the SCPTS modules and sgRNAs, then quantified *SHH* messenger RNA after 24 hours photostimulation. Guide 1 was the most efficient (800-fold *SHH* expression over a nontargeting guide; Extended Data Fig. 3b). In addition, we designed guides for another morphogen involved in neurodevelopment, *BMP4*: guide 3 was the most effective (Extended Data Fig. 3c,d). Expression of these targets in dark controls was approximately 5% of the lit for *SHH* guide 1 and 30% for *BMP4* guide 3. We note that *BMP4* is natively expressed in HEK cells, which is the reason for the lower induction and higher expression in the control, of which approximately half (roughly 15%) can be attributed to leakage (Extended Data Fig. 3d). We assessed whether the induced *SHH* exerted its biological activity by stimulating the expression of known downstream genes in human induced pluripotent stem cells (hiPSCs). We measured *FOXA2*, *FOXG1*, *NKX2-1*, *NKX6-2* and *OLIG2* expression after 24–72 hours of stimulation. *SHH* reached its highest level at 24 hours and decreased at 48 and 72 hours (Extended Data Fig. 3e,f). Within 72 hours, *FOXA2*, *FOXG1*, *NKX6-2* and *OLIG2* were significantly upregulated in neural induction media, but not in stem cell media, despite *SHH* being highly induced in both conditions (Extended Data Fig. 3e,f). In parallel, we used the PA-Cre–Lox system to generate a stable hiPSC line that overexpresses a NeonGreen-SHH cassette on light stimulation and doxycycline treatment^5^. With this system, we observed stronger *SHH* mRNA expression but also higher background levels due to roughly 2–3% leakage, and the same was true for its downstream regulated genes (Extended Data Fig. 3e,g).

**Fig. 3:**
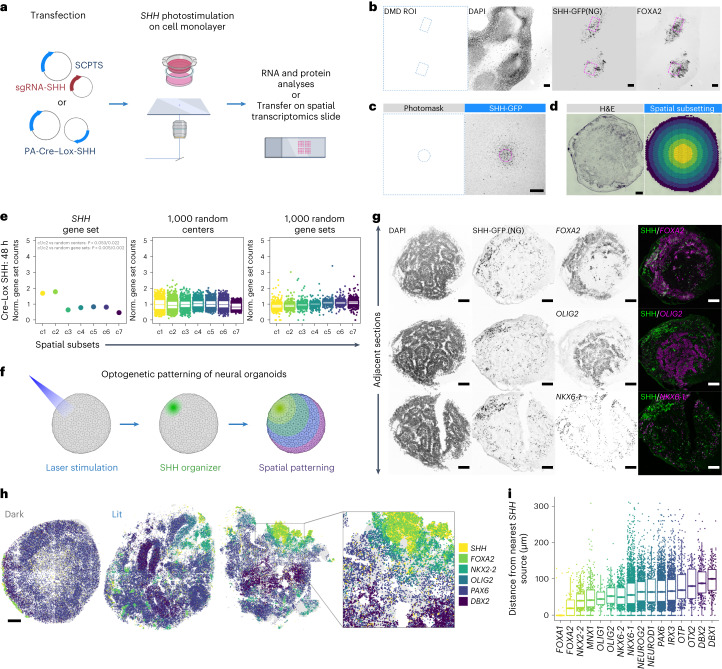
Optogenetic stimulation of *SHH* in human stem cells and organoids. **a**, Optogenetic stimulation of *SHH* coupled with spatial readouts. **b**, Representative (*n* = 3) imaging of DAPI, SHH-expressing cells (NG) and FOXA2 (immunofluorescence) in hiPSCs 6 days poststimulation. *SHH* was induced in two ROI (left and in magenta) with a DMD. Scale bars, 100 μm. **c**, Representative (*n* = 3) image of hiPSCs Cre–Lox *SHH* cultured on a PET membrane, stimulated through a 500 μm-wide circular photomask (left). The right shows NG. Scale bar, 500 μm. **d**, The left shows representative (*n* = 4) hematoxylin and eosin (H&E) staining of a hiPSC layer cultured on a membrane and transferred onto a Visium slide. The right shows spatial subsetting of Visium spots into seven concentric circles, centered on the SHH-induced area. Scale bar, 500 μm. **e**, The left shows normalized (Norm.) counts of an *SHH* gene set in the seven concentric circles (c1–7), color coded as in **d**, in hiPSCs stimulated for 48 h (*n* = 1; other samples in Extended Data Fig. 3). The middle shows the same as the left, sampling 1,000 times a random spot as center. The right is the same as the left, sampling 1,000 times a random gene set. Exact *P* < 0.05 are indicated, computed as the fraction of values exceeding the tested value from random sampling of 1,000 centers and 1,000 gene sets. **f**. Optogenetic patterning of neural organoids. **g**, Representative (*n* = 5) imaging of DAPI, SHH-expressing cells marked by NG and *FOXA2/OLIG2/NKX6-1* in adjacent cryo-sections of neural organoids with laser induction of *SHH* in the north-west pole. The signal is in gray scale for each target separately, and merged in green and magenta (right). Scale bars, 100 μm. The experiment was performed four times and the representative images are shown here. **h**, Molecular Cartography spatial transcriptomic data of control (left, dark) and SHH-induced (right, lit) organoids, with the indicated transcripts colored according to the legend (right). Experiment was performed four times per condition and three examples are shown here (one dark and two lit). Scale bar, 100 μm. **i**, Distance distribution (μm) of cells expressing the indicated transcripts from the nearest SHH^+^ cell, in the most left induced organoid of **h**. In all boxplots, the center and bounds represent the median, 25 and 75% quantiles. Whiskers, if present, represent 1.5× interquartile ranges.

We then performed localized *SHH* activations in two dimensions (Fig. 3a,b and Supplementary Video 1): robust expression of *FOXA2* was visible at the protein level after inducing *SHH* for 6–7 days in defined ROI with the DMD setup. To systematically profile spatial gene expression on local gene perturbations, we used sequencing-based spatial transcriptomics on cultured cells (10X Visium). We adapted hiPSC culture and photostimulation to cells cultured on a polyester (PET) hanging insert. The insert membrane is cut from its scaffold, transferred onto a slide and removed after cell fixation (Fig. 3a,c and Extended Data Fig. 3h,i). We used this system to probe the gene expression response to the induction of *SHH* in the center of the membrane for a time course of 120 hours, with the PA-Cre–Lox system. Since the RNA capture was not homogeneous, yielding vastly different unique molecular identifier (UMI) counts across the capture area (Fig. 3d and Extended Data Fig. 3j), we merged the transcript counts for a set of concentric circles, with the inner circle enclosing the photostimulated area (Fig. 3d). We examined a gene set comprising *SHH* and its targets, and retrieved a peak of expression in the inner parts of the membrane for all time points compared to randomized controls (48 h in Fig. 3e and other time points in Extended Data Fig. 3k, l). We note that the raw molecule counts for these transcripts were low (globally, in the range of tens or hundreds), and dominated by *SHH* at the earliest time point. When considering additional genes in the pathway, we found that the receptor *PTCH1* was strongly upregulated in proximity to *SHH* at 120 hours, as, to a lesser extent, *SMO* (Extended Data Fig. 3m). Finally, we ruled out the activation of a heat shock transcriptional program (Extended Data Fig. 3n).

### Optogenetic patterning of neural organoids

**Extended Data Fig. 4:**
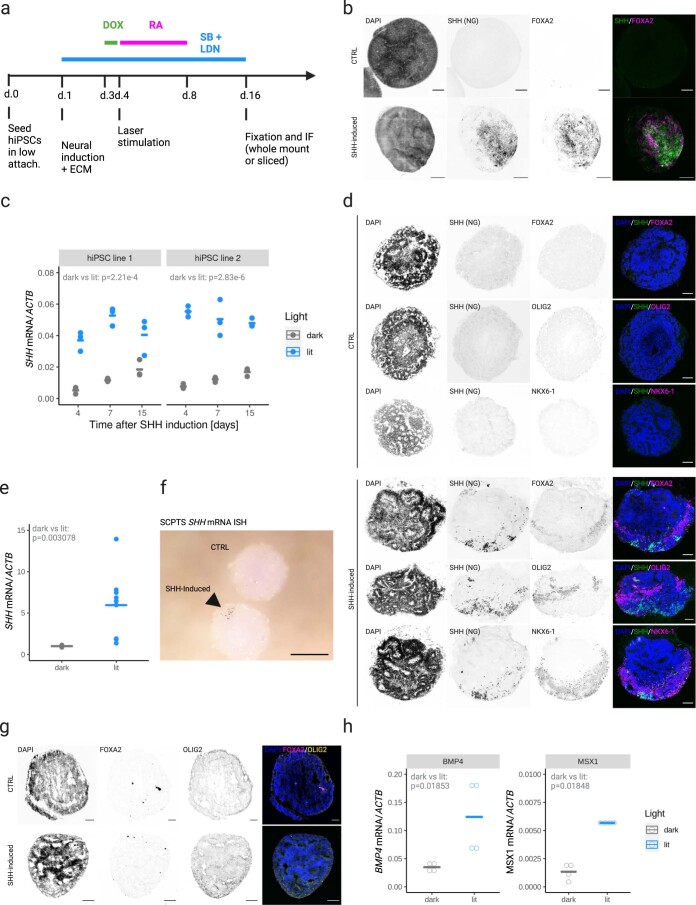
Optogenetic stimulation of Sonic Hedgehog in human organoids. **a**. Schematic representation of neural organoids protocol. **b**. Representative (n = 3) DAPI, SHH-NeonGreen and *FOXA2* (immunofluorescence) in whole-mount organoids without and with localized laser induction. Signal is shown separately in grey scale for each target and merge for *SHH* and *FOXA2* in green and magenta (right). Scale bar: 100 μm and 500 μm for non-induced and induced organoids respectively. **c**. RT-qPCR measurement of *SHH* mRNA levels in PA-Cre/Lox-SHH organoids derived from two hiPSC lines (left and right). Relative expression of *SHH* over *ACTB* is shown for dark and lit conditions (grey and blue) at different times. Each dot represents a biological replicate (pool of 3–6 organoids, n = 3), horizontal bars indicate their mean. Benjamini-Hochberg corrected two-sided t test p-values shown on top. **d**. Representative (n = 3) DAPI, SHH-NeonGreen, FOXA2, OLIG2 and NKX6-1 immunofluorescence in cryosectioned organoids from hiPSC line 2, without induction (top) and laser induction (bottom three rows). Signal is shown separately in grey scale for each target and merged in blue, green and magenta (right). Scale bar: 100 μm. **e**. RT-qPCR measurement of the *SHH* mRNA levels in a stable SCPTS-SHH hiPSC line. Relative expression of *SHH* over *ACTB* is shown for dark and lit conditions at 48h post- stimulation. Each dot represent a biological replicate (pool of 3–6 organoids, n = 8 dark, 9 lit), horizontal bars indicate their mean. P-value (two-sided t test) shown on top. **f**. Representative (n = 2) *SHH* mRNA *in situ* hybridization for non-induced and SHH locally induced (arrow) organoids at 48h post-stimulation. Scale bar: 500 μm. **g**. Representative (n = 3) Immunofluorescence of DAPI, FOXA2 and OLIG2 in cryosectioned organoids from the SCPTS hiPSC line, without (top) and with laser induction of *SHH* in a pole (bottom). Signal is shown separately in grey scale for each target and merged in blue, yellow and magenta (right). Scale bar: 100 μm. **h**. *BMP4* (left) and *MSX1* (right) mRNA RT-qPCR measurement, normalized on *ACTB*, in neural organoids derived from a PA-Cre/Lox-BMP4 hiPSC line, dark vs lit at 4 days post-photostimulation. Each dot represent a biological replicate (pool of 3–6 organoids, n = 4), horizontal bars indicate their mean. Benjamini-Hochberg corrected two-sided t test p-values are shown on top. Source data

*SHH* induction produced a biological response in hiPSCs only detectable with sensitive techniques in short time intervals (Fig. 3e and Extended Data Fig. 3e,f), becoming more robust over time (with FOXA2 becoming detectable 6–7 days poststimulation at the protein level; Fig. 3b). To overcome the constraint of a 2D system that has limited endurance and poor physiological resemblance to a developing tissue, we devised a protocol for producing 3D neural organoids, partially based on previous attempts at mimicking the DV patterning of the caudal part of the neural tube in vitro^12^. Besides the higher basal expression of the transgene, we chose to use the PA-Cre–Lox system for this application since a single, short photostimulation allows for sustained and prolonged *SHH* expression, and *SHH*-producing cells can be tracked with a fluorescent tag. We used laser scanning to activate *SHH* in a pole of embryoid bodies grown for 4 days (Figs. 2g–j and 3f and Supplementary Video 2), then supplemented the medium with retinoic acid for 5 days to induce a posterior fate, and allowed them to grow and differentiate for an additional 7 days (Extended Data Fig. 4a).

We tracked the fluorescent tag to assess spread and location of *SHH*-expressing cells in whole-mount fixed organoids, and noticed that some exhibited significant spread away from the induced pole, likely due to cell divisions and migration. Nevertheless, the organoids retained an overall polarized *SHH* expression as previously observed with live imaging (Fig. 2j), which induced robust and spatially restricted activation of *FOXA2*, whereas noninduced organoids produced neither detectable *SHH* nor *FOXA2* at the protein level (Extended Data Fig. 4b). We stained consecutive organoid cryogenic (cryo)-sections for *SHH* targets known to be induced in different neural tube domains at increasing distance from the *SHH* source^9^ and observed that FOXA2, OLIG2 and NKX6-1 established diverse spatial expression domains (Fig. 3g).

In a second, genetically distinct hiPSC line, *SHH* expression was induced at the RNA level to the same extent (Extended Data Fig. 4c) and FOXA2, OLIG2 and NKX6-1 again exhibited patterned expression (Extended Data Fig. 4d). As previously hypothesized, robust activation of these ventral marker genes required strong and prolonged *SHH* expression. In fact, only a transient (up to 24 hours) activation of the endogenous *SHH* locus with the SCPTS system was sufficient to induce SHH expression up to 48 hours poststimulation, but not enough to achieve robust patterning at day 12 (Extended Data Fig. 4e–g). Finally, we also generated a PA-Cre–Lox hiPSC line for light-inducible activation of a *BMP4* cassette, and found that 24 hours of photostimulation were sufficient to induce the already high *BMP4* mRNA expression, which in turn upregulated the expression of the dorsal marker *MSX1* of roughly fourfold after 4 days (Extended Data Fig. 4h).

**Extended Data Fig. 5:**
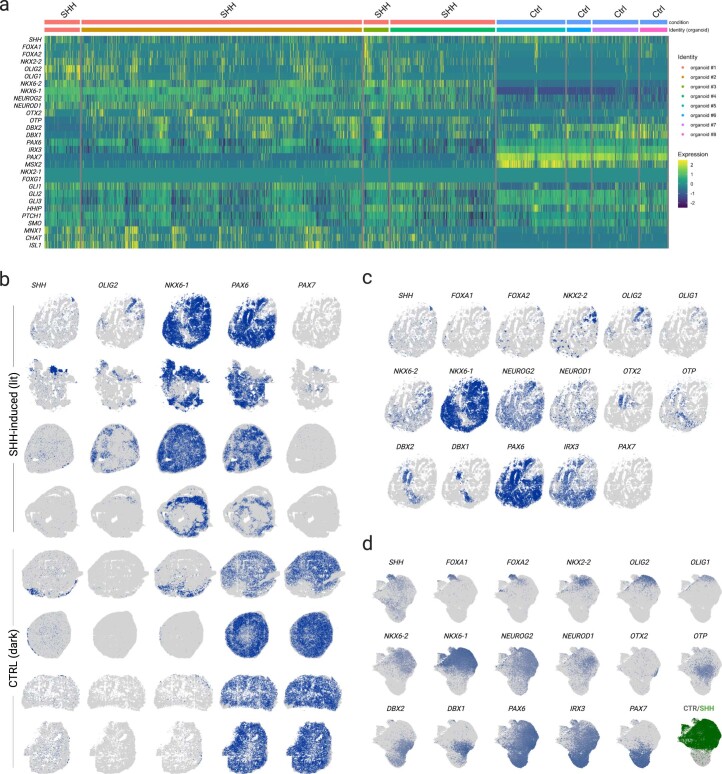
Spatial transcriptomic analysis of control and SHH-induced organoids. **a**. Heatmap of log-normalized transcript expression for a panel of transcripts of interest involved in the *SHH* pathway, quantified by Molecular Cartography spatial transcriptomics, for 8 organoids (4 controls and 4 SHH-induced as indicated on top). **b**. Molecular Cartography signal of selected transcripts of interest overlaid on a grey mask of a representative cryosection for each organoid (4 controls/dark and 4 SHH-induced/lit). **c**. Molecular Cartography signal of more selected transcripts of interest overlaid on a grey mask of a representative SHH-induced organoid cryosection. **d**. UMAP plot of log-normalized and scaled expression of the same transcripts as in e. for all detected cells in the 8 organoids. Lower right: organoids condition (control and SHH-induced in grey and green).

To more comprehensively characterize the spatial patterning activity of *SHH*, we optimized organoid embedding and fixation for FISH-based spatial transcriptomics (Molecular Cartography, Resolve Biosciences) and imaged a panel of 88 transcripts, including known *SHH* targets and markers for distinct DV neural tube cell populations. We performed such analysis on four control and four SHH-induced organoids: while control organoids displayed some surface expression of *SHH* and a few of its targets, photostimulated organoids were strongly patterned into distinct spatial gene expression territories (Fig. 3h). More ventral markers (*NKX2-2*, *OLIG1/2*, *NKX6-1/2*, *DBX1/2*) were induced on *SHH* activation, while exclusively dorsal markers such as *PAX7*, *MSX2* were almost completely depleted (Extended Data Fig. 5a,b).

In these experiments, the cryo-sectioning plane cannot be controlled to intersect the photostimulation plane, nor can sections in which this is the case be selected for subsequent analysis, since fixation quenches the NeonGreen signal. However, we generally observed that DV positional marker genes were expressed in spatially distinct territories (Fig. 3h and Extended Data Fig. 5b,c). Moreover, when the cryo-sectioning plane clearly intersected the photostimulation plane (Fig. 3h, right panel), the spatial distribution of these transcripts directly resembled that of the neural tube DV axis, consisting of mutually exclusive territories defined by the expression of single or combined markers expressed at defined distances from the *SHH* source (Fig. 3h,i and Extended Data Fig. 5b,c). For example, *FOXA1/2*, *NKX2-2* and *OLIG2* expression domains were depleted of *PAX6* and *IRX3*, while *DBX1*, *DBX2*, *OTP* and *OTX2* expression was confined to spatially distinct regions farther away (Extended Data Fig. 5c). We found that *FOXA1* and *FOXA2* expression patterns differed as *FOXA1* was mostly confined to *SHH*^+^ cells and *FOXA2* was also spread in the immediate vicinity in a noncell autonomous manner (Fig. 3i). Overall, these features are observable in the physical space of single organoid sections (Extended Data Fig. 5c), and captured by dimensionality reduction of the gene expression space of 43,230 segmented cells from eight organoids (Extended Data Fig. 5d).

From these data, we conclude that localized activation of *SHH* signaling can induce spatially restricted patterns of RNA expression in neural organoids, marked by genes known to specify distinct populations of progenitor cells in vivo and resembling their spatial relationships in the vertebrates’ neural tube.

### Effects of localized *SHH* activation on organoids’ gene expression

From the previous experiments, we concluded that optogenetic activation of *SHH* in neural organoids induced the expression of known marker genes in distinct spatial territories. The global effects of *SHH* activation on gene expression and its resemblance to in vivo development, however, remained unclear.

**Fig. 4:**
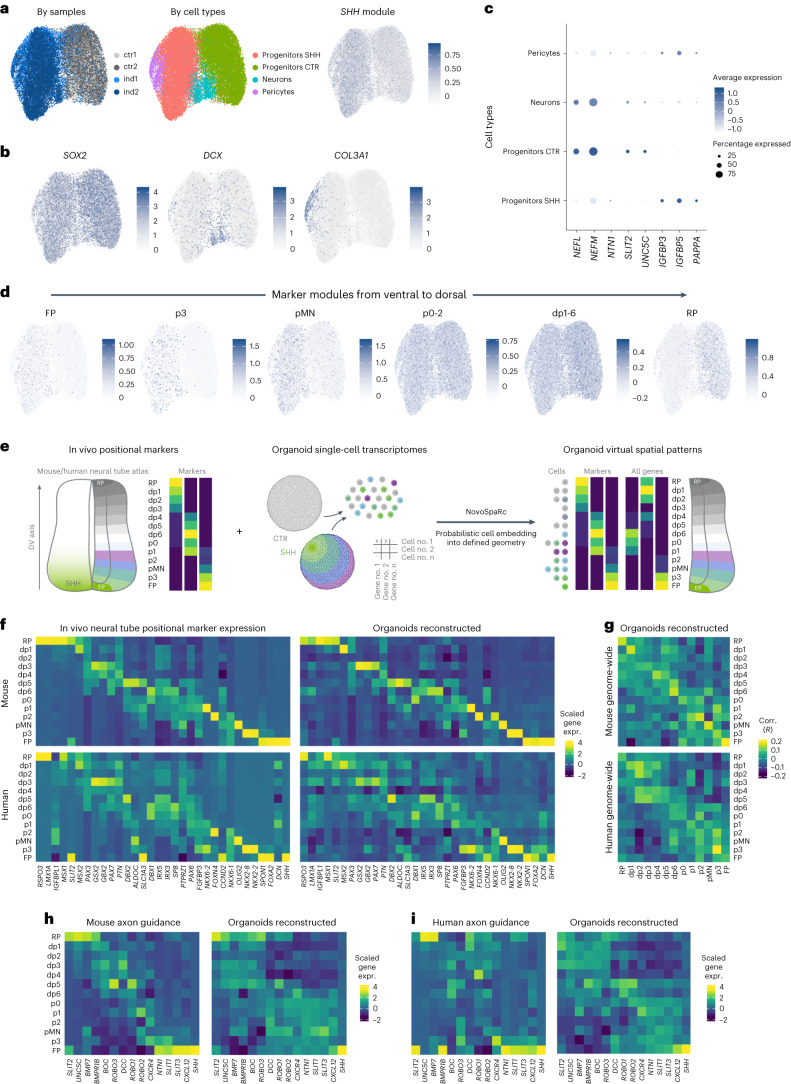
Molecular effects of *SHH* and spatial gene expression patterns in neural organoids. **a**, Sample identity (left), annotated cell types (middle) and *SHH* module score (right) from two replicates of control and *SHH*-induced organoids scRNA-seq data (UMAP). **b**, Featureplot of log-normalized expression of markers for main cell types. **c**, Heatmap of log-normalized and scaled expression for selected examples of differentially expressed genes. **d**, Featureplot of module scores for marker genes of ventral (floor plate, FP) to dorsal (roof plate, RP) domains of gene expression of the mouse developing caudal neural tube. **e**, NovoSpaRc reconstruction of DV identities in organoids single-cell transcriptomic data. The left shows a set of positional marker genes is selected from neural tube sRNA-seq data. The middle shows that control and SHH-induced organoids single-cell transcriptomes are merged, and the right shows cells are probabilistically embedded into a DV geometry and genome-wide gene expression is reconstructed. **f**, The left shows the scaled gene expression of positional marker genes in mouse (top) and human (bottom) scRNA-seq data. The right shows the same in organoid reconstruction based on mouse (top) or human (bottom) expression data. **g**, Correlation matrix for mouse (top) and human (bottom) versus organoid reconstructed DV gene expression domains, computed on 1,000 highly variable genes subsequently filtered for expression in each dataset. **h**, Scaled (*z*-score) expression of a set of genes involved in axon guidance in progenitor cells of the mouse developing spinal cord and in the organoids spatial reconstruction. **i**, As **h**, for the reconstruction based on human scRNA-seq data.

**Extended Data Fig. 6:**
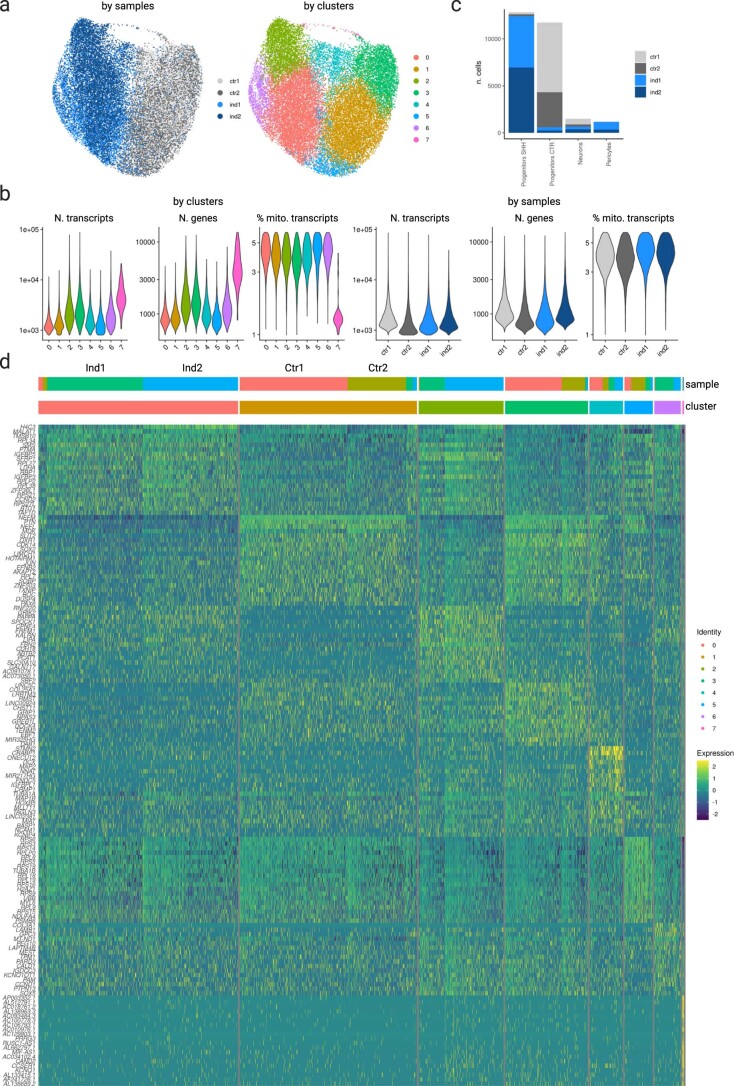
Single-cell analysis of control and SHH-induced organoids. **a**. UMAP representation of all sequenced cells with > 800 UMIs and < 5% mtRNA, before filtering out two low-quality clusters (5 and 7). Left: samples of origin (controls in grey, induced in blue); right: identified clusters. **b**. Distribution of transcripts and gene counts and mtRNA % by clusters and samples. **c**. Cell number by cell type and sample. **d**. Heatmap of top 20 marker genes expression for all clusters.

**Extended Data Fig. 7:**
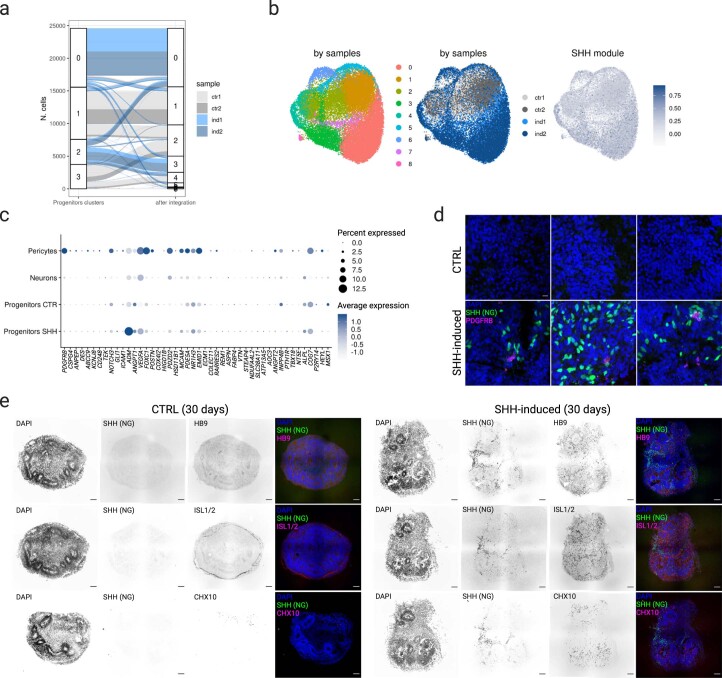
Single-cell analysis and validations of control and SHH-induced organoids. **a**. Alluvial plot showing the relative composition of the progenitor cells clusters in control (grey) and SHH-induced organoids (blue), before and after data integration (see Methods). **b**. Sample identity (left), annotated cell types (middle) and *SHH* module score (right) from 2 replicates of control and SHH-induced organoids scRNA-seq data (UMAP). Same as Fig. 4a, but after data integration (see Methods). **c**. Dotplot showing % of cells expressing and log-normalized, scaled gene expression for known marker genes of pericytes. **d**. Representative (n = 3) immunofluorescence images for DAPI, NeonGreen (SHH tag) and PDGFRB (pericyte marker) in control and SHH-induced organoids at 12 days post-photo-stimulation. Experiment was performed in 4 replicates, 3 representative FOVs are shown. Scale bar: 10 μm. **e**. Representative (n = 3) immunofluorescence of DAPI, NeonGreen (SHH tag) and HB9, ISL1/2 and CHX10 in control (left) and SHH-induced organoids at 30 days post-photo-stimulation. Signal is shown separately in grey scale for each target and merged for SHH and the protein of interest in green and magenta (right). Scale bar: 100 μm.

To address these questions, we performed scRNA-seq of control and *SHH*-induced organoids. After normalization, dimensionality reduction, quality filtering and clustering (Extended Data Fig. 6a,b), two small artifactual clusters were removed and the remaining transcriptomes (12,463 from controls and 14,833 from induced organoids) were categorized into four main cell types: two clearly distinct *SOX2*^+^ neuronal progenitor populations from control and *SHH*-induced organoids, the latter marked by increased expression of *SHH* and its targets, *DCX*^+^ neurons in similar numbers from both conditions, and an additional cluster specifically present in *SHH*-induced organoids and marked by genes encoding extracellular matrix components such as *COL3A1*, *LAMB1* and *GPC3* (Fig. 4a,b and Extended Data Fig. 6c,d; additional controls in Extended Data Fig. 7a,b). It has been observed that differentiation of oligodendrocyte progenitors can yield a number of *COL3A1*^+^ pericyte-like cells^13,14^, and our data indicate that our perturbation may specifically stimulate a related differentiation trajectory. By looking at pericyte markers, we found that many of them were enriched in these cells (Extended Data Fig. 7c), and we observed PDGFRB^+^ cells in the proximity of SHH-producing cells by immunofluorescence (Extended Data Fig. 7d). At a later stage (30 days poststimulation), we also validated the expression of HB9/ISL1 and CHX10, markers for motor neurons and V2A interneurons deriving from ventral progenitor populations (Extended Data Fig. 7e).

**Extended Data Fig. 8:**
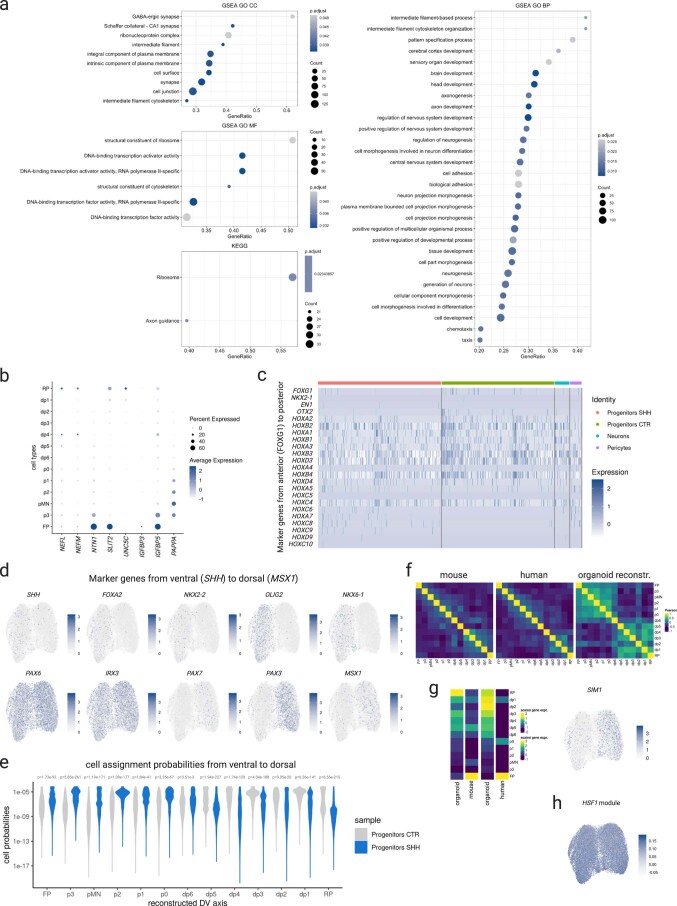
Molecular effects of SHH and spatial gene expression patterns in neural organoids. **a**. GSEA analysis on GO CC, MF and BP terms, and on KEGG pathways, performed on a rank list of differentially expressed genes between control and induced progenitors. P-values: Benjamini-Hochberg-corrected GSEA test. **b**. Dotplot of exemplary differentially expressed genes in CTR vs SHH organoids, for human neural tube scRNA-seq data. **c**. Heatmap of HOX genes and additional anterior-posterior markers showing that control and induced organoids have hindbrain/spinal cord identity, marked by expression peak between *HOXB2* and *HOXC4*. **d**. Featureplot of log-normalized expression of a set of positional marker genes along the ventral (*SHH*)-dorsal(*MSX1*) axis. **e**. Assignment probabilities of progenitor cells from control and induced organoids to each of the 13 progenitor domains specified by positional markers of the mouse neural tube atlas. P-values (Benjamini-Hochberg-corrected Wilcoxon Mann Whitney) for the comparison between control and SHH-induced organoids are indicated on top for each DV domain. **f**. Correlation matrices for mouse, human and organoids reconstructed DV gene expression, computed on 1000 highly variable genes filtered for expression in each dataset. **g**. Left: DV scaled (z-score) expression of *SIM1* from organoid reconstructed and mouse/human DV domains. Right: featureplot of log-normalized expression of *SIM1* in organoids. **h**. Featureplot of module score for a gene module comprised of the heat shock transcription factor HSF1′s targets.

By comparing gene expression in control versus *SHH*-induced progenitors (Fig. 4c and Extended Data Fig. 8a), we found strong changes in genes and gene sets involved in shaping the neuronal cytoskeleton (for example, *NEFL* and *NEFM*), axon guidance (for example, *NTN1*, *UNC5C* and *SLIT2*), and regulators of the IGF pathway (*IGFBP3*, *IGFBP5* and *PAPPA*). The up- or down-regulation of these genes in SHH-induced organoids is matched by a clear DV polarization of their expression in vivo (Extended Data Fig. 8b).

To further compare the sequenced cells and in vivo neural tube in terms of positional identities, we first considered the expression of HOX genes and additional markers of the rostrocaudal axis^15^, which indicated that the sequenced cells bear similarity with the posterior part of the hindbrain and the anterior part of the developing spinal cord (Extended Data Fig. 8c), with the exception of *OTX2*, a forebrain and midbrain marker that was expressed in a narrow and spatially distinct subset of cells (Extended Data Figs. 5c and 8c). We then compared known positional marker genes along the DV axis of the mouse and human developing spinal cord^16,17^ for its 13 distinct domains, from the most ventral floor plate, 11 progenitor domains (p3, pMN, p0-2, dp1-6), to the roof plate dorsally. In concordance with *SHH*’s known role, we observed a clear ‘ventralization’ of cells in *SHH*-induced organoids. In comparison, the most dorsal markers of the neural tube were largely confined to noninduced organoids, while markers for intermediate domains were expressed in both conditions (Fig. 4d and Extended Data Fig. 8d), as previously observed by spatial transcriptomics.

Given that marker genes for all 13 domains were expressed in induced, control or both conditions, we reasoned that by merging all cells we may be able to reconstruct DV gene expression patterns genome wide. To do so, we used novoSpaRc^18^, which uses optimal transport to probabilistically embed cells into a predefined geometry—in this case the neural tube DV axis—under the assumption that differences in gene expression are to be locally minimized (that is, adjacent domains are more similar than domains farther away) and anchored to the known expression of positional markers (Fig. 4e). By informing novoSpaRc with a set of 32 positional markers retrieved from a mouse^16^ and a human developing spinal cord atlas^17^, we were able to obtain a similar DV pattern in the human organoids data (Fig. 4f), with cells from induced organoids preferentially assigned to domains from the floor plate to dp6 and cells from controls from dp5 to the roof plate (Extended Data Fig. 8e). This was in accordance with the spatial transcriptomics data, which showed *PAX7* and *MSX2*, expressed dorsally to dp6 in vivo, were almost completely depleted in induced organoids (Extended Data Fig. 5a).

When comparing this reconstruction with global mouse and human in vivo data, we observed a signal along the diagonal of a correlation matrix across the DV domains (Fig. 4g and Extended Data Fig. 8f). However, the magnitude of this correlation was low, suggesting, as we could expect, that in vitro modeling of neural tube development can recapitulate certain gene expression programs observed in vivo but also presents substantial differences. Nonetheless, our reconstruction allowed to examine genes regulated along the in vitro DV axis and to validate their expression pattern with in vivo data. We observed that the genes encoding axon guidance determinants^19^ in the developing spinal cord were strongly polarized along the reconstructed DV axis as they are in vivo (Fig. 4h–i). However, we also observed remarkable differences; for example, the transcription factor *SIM1*, which is expressed ventrally in the developing spinal cord and hypothalamus, was instead enriched dorsally along our reconstructed DV axis, and was in fact down-regulated in SHH-induced organoids (Extended Data Fig. 8g). Finally, for these data, we also ruled out unwanted activation of heat shock genes (Extended Data Fig. 8h).

## Discussion

In animals, the generation of complex body plans from a single cell involves an orchestrated series of patterning events arising from the definition of spatially restricted gene expression territories during development. To study these phenomena, we need a means to chart gene expression in tissue space with high sensitivity and resolution, and models for reproducing and interrogating the regulatory principles behind them.

In the past few years, spatial investigation of gene expression has made enormous advances, with new technologies for high-throughput spatial transcriptomics^20^. Meanwhile, in the field of stem cell-based tissue modeling, the use of 3D organoid culture has gained great interest for its capability to reproduce fundamental aspects of development in vitro^2^. One limitation of these technologies is in the ability to control tissue patterning, as titrating the concentration of signaling molecules in the culture media can generate asymmetries, but these are not programmable^21–23^. A practicable solution is to produce organoids with an engineered organizer, which has been achieved, for example, by letting forebrain organoids aggregate onto a core of cells producing the morphogen SHH in a doxycycline-dependent manner^24^. Here, we reported an alternative method based on optogenetic perturbations of gene expression, combined with single-cell and spatial transcriptomic readouts.

Spatial gene perturbations may be useful in studying numerous biological processes in organoids, especially those involving cell–cell communication or the generation of gene expression asymmetries in tissues. These methods might be applied, for example, to control oncogenes in targeted single cells within organoids or tumoroids^25^ to study the interplay of the transformed cells with their neighborhood over time. On the other hand, locally perturbing cytokines involved in immune signaling and then studying their local impact on the surrounding tissue, or studying axon guidance by locally activating diffusible signals and then tracking their effects on neuronal connectivity, could be further enabled in different types of organoid by a spatially programmable gene perturbation system. Finally, an interesting application is the study of developmental processes by controlling signaling pathways to study patterning in a simplified setup such as cerebral or spinal cord organoids, where the position of sender or receiver cells are programmed by photostimulation^26^.

In conclusion, we believe that combining optogenetics with spatial transcriptomics might prove extremely useful for generating and characterizing new organoid models with complex and controlled spatial patterning modalities, for studying spatiotemporal mechanisms of morphogen signaling in particular, or gene regulation in general, and to study disease-relevant genes involved in cell–cell communication. On the other hand, we stress that limitations of this approach, including some degree of leakage, the lower efficiency of knock-downs with respect to gene activations and the difficulty in combining precise photostimulations with 3D models where cells actively proliferate and migrate over time, will require further technical improvements.

## Methods

### Cell culture, transfections and cell line generation

HEK293T cells were cultured in DMEM (high-glucose, with glutamax and pyruvate, Thermo Fisher, catalog no. 11360872) supplemented with 10% Tet-free FBS (PAN Biotechnology, no. P30-3602) in absence of antibiotics at 37 °C with 5% CO_2_. They were split every 2 or 3 days with 0.05% trypsin in 10 cm cell culture dishes. We note that HEK293T cells appear in the ICLAC register of commonly misidentified cell lines. We used these cells for transfection experiments as is common practice in the field. For transfection experiments shown in Supplementary Fig. 2, 30,000 cells were seeded the evening before transfection in 70 μl of medium on white 96-well, clear-bottom plates (Corning, catalog no. 3610). The morning after, a mix composed of 12 μl of Optimem (Thermo Fisher, catalog no. 31985062), 25 ng Luciferase-encoding plasmid, 150 ng guide RNA-encoding plasmid and 150 ng of Cas13-encoding plasmid or 2× 100 ng of each Split-Cas13-encoding plasmid was mixed with 25 μl of Optimem and 0.5 μl of Lipofectamine 2000 (Thermo Fisher, catalog no. 11668019), incubated at room temperature for 10 min and then pipetted onto the cells. Light stimulation was started 6 h posttransfection and luciferase assay was performed in the same plate 24 h postinduction by removing 50 μl of medium, adding 75 μl of luciferase assay buffer (Promega Dual-Luciferase Reporter Assay System, catalog no. E1910), incubating for 10 min at room temperature, before reading Firefly luciferase, adding 75 μl of Stop&Glo buffer, incubating for 10 min at room temperature and then reading the Renilla luciferase. Plate readings were performed in a Tecan M200 infinite Pro plate reader with 2 s of integration for luciferase measurement using i-control (v.1.1) software.

For transfection experiments shown in Figs. 1 and 2 and Supplementary Figs. 2 and 3, 25,000 cells were seeded the evening before transfection in 70 μl of medium on black 96-well, clear-bottom plates (Corning, catalog no. 3904). The morning after, a transfection mix composed of 25 μl of Optimem, 0.4 μl of P3000 and 100–300 ng of plasmid DNA was pooled with 25 μl of Optimem and 0.3 μl of Lipofectamine 3000 (Thermo Fisher, catalog no. L3000001), incubated for 10 min at room temperature and then pipetted onto the cells. Light stimulation was started 6 h posttransfection and live-cell imaging was performed at 24 h postinduction unless differently indicated (for example, for the time course in Fig. 1).

Transfections for the RNA-seq and proteomics experiments shown in Supplementary Fig. 2 were performed in six-well plates with 1 million HEK293T cells per well. The morning after seeding, a mix composed of 250 μl of Optimem, 800 ng of ePB Puro TT RFP plasmid, 1,000 ng of guide RNA-encoding plasmid and 1,000 ng of Cas13-encoding plasmid, 8 μl of p3000 reagent and 2 μl of doxycycline (1 mg ml^−1^) was mixed with 250 μl of Optimem and 6 μl of Lipofectamine 3000, incubated at room temperature for 10 min and then pipetted onto the cells. Cells were gathered 36 h posttransfection for RNA extraction with home-made Trizol or for protein purification, as described later in the Bulk RNA-seq and LC–MS proteomics sections.

Transfections for generating the Cre–Lox CasRx line were performed in 12-well plates seeded with 100,000 HEK293T cells and transfected the day after with 250 ng of ePB-PA-Cre plasmid, 500 ng of LoxP-CasRx plasmid, 125 ng of hyperactive transposase plasmid, 100 μl of Optimem and 2.5 μl of p3000 reagent, mixed with 100 μl of Optimem and 1.5 μl of Lipofectamine 3000 reagent, incubated for 10 min at room temperature and then pipetted onto the cells. After 3 days posttranfection, cells were split into two wells of a new six-well plate and selected with puromycin (1 μg ml^−1^) and blasticidin (5 μg ml^−1^) for 1 week. RFP^+^/GFP^−^ cells were further sorted by FACS (fluorescence-activated cell sorting) to increase the purity of the population.

HiPSCs (hiPSC line no. 1: XM001, ref. ^27^, and hiPSC line no. 2: Gibco Human Episomal iPS line 1E6, catalog no. A18944, lot no. 2036936) were obtained by the BIMSB MDC Organoid platform. They were cultured in E8 flex media with supplement (Thermo Fisher, catalog no. A2858501) at 37 °C in hypoxia (5% O_2_) conditions, passaged every 3–4 days with accutase and seeded on Geltrex- (Gibco catalog no. A1413302) coated plates. To promote their attachment to the plates, cells were kept in E8 flex media supplemented with 10 μM Rho-associated protein kinase (ROCK) inhibitor. Media was changed to E8 flex without ROCK inhibitor within the next 24 h from plating.

The PA-TetON CasRx cell line was produced with two lentiviruses produced in HEK293T cells with the PA-TetON plasmid^4^ and the TRE-CasRx plasmid, two packaging plasmids (Addgene catalog nos. 8454 and 8455). Next, 50,000 HEK293T cells were transduced with a multiplicity of infection of 10 of each lentivirus in a 24-well format and then selected for blasticidin expression (5 μg ml^−1^) for 1 week.

HiPSCs transfections were conducted using Lipofectamine Stem (Thermo Fisher catalog no. STEM00001). For transfection experiments, regarding the time course of SHH activation shown in Fig. 3 and Extended Data Fig. 2, hiPSC colonies at 70–80% confluency were dissociated to single cells with accutase. Next, 250,000 cells were then resuspended in 100 μl of E8 flex with 10 μM ROCK inhibitor and seeded on black 96-well plates, previously coated with Geltrex. After 2–3 h, when cells got attached to the wells, media was changed with 50 μl of E8 flex with ROCK inhibitor. The transfection was performed as follows: for one sample, 1.2 μl of Lipofectamine were mixed with 25 μl of Optimem and 500 ng of total plasmids were diluted in 25 μl of Optimem. Diluted Lipofectamine and diluted plasmids were then combined at a ratio of 1:1, incubated at room temperature for 10 min and 50 μl pipetted drop by drop on top of the cells. Transfection efficiency was assessed by including a control plasmid encoding for constitutive GFP (pmax-GFP, Lonza, catalog no. V4YP-1A24). Media was changed within 7–8 h after transfection to E8 flex and, before light induction, gradually replaced by neural induction media ‘COM1’, whose composition is as follows: DMEM-F12 (Thermo Fisher, catalog no. 11320033), N2 supplement (Thermo Fisher, catalog no. 17502048), Neurobasal (Thermo Fisher, catalog no. 21103049), B27-vitamin A (Thermo Fisher, catalog no. 12587010), 1× penicillin–streptomycin, Glutamax (Thermo Fisher, catalog no. 35050061), 2-mercaptoethanol, vitamin C, chemically defined lipid concentrate and insulin. The reason of this media switch was due to the fact that E8 flex contains basic fibroblast growth factor (FGF2), which is a strong inhibitor of the SHH signaling pathway^28^. Light stimulation was started within 15 h posttransfection, using a blue LED array. Cells were gathered after each time point of light stimulation and lysed with 100 μl of home-made Trizol. RNA was then extracted with the Zymo RNA extraction kit (Zymo catalog no. R2051). Complementary DNA (cDNA) preparation and quantitative PCR with reverse transcription (RT–qPCR) were performed as described in the section RNA extraction with RT–PCRs.

The modules in the SCPTS2.0 system^3^ (split Cas9, MCP, sgRNA cassette) were resynthesized and PCR-amplified, then recloned into two lentiviral plasmids (pLKO.1 neo, Addgene, catalog no. 13425; pLJM-EGFP, Addgene, catalog no. 19319) and, from the latter, they were subcloned into two PiggyBac transposons vectors. All stable hiPSCs (PA-Cre–Lox-SHH, PA-Cre–Lox-BMP4, PA-Cre–Lox CasRx and SCPTS-SHH) lines were generated by transfecting hiPSCS with PiggyBac vectors and a plasmid encoding a hyperactive PiggyBac transposase. In this regard, 400,000 hiPSCs were plated in a Geltrex-coated well of a 12-well format plate and transfected with Lipofectamine Stem. Herein, 400 ng of each transposon—one carrying the ‘TRE-CRE split1 nMag -T2A/P2A- pMag CRE split2’ and the other carrying the LoxP cassettes—were combined with 200 ng of hyperactive transposase and diluted in 72 μl of Optimem. Diluted DNA was then mixed with Lipofectamine Stem (3 μl), also diluted with Optimem (72 μl) and incubated at room temperature for 10 min. Media was changed to E8 flex after 5–12 h from transfection. Cells were left to recover for 4 days and the antibiotics selection began, immediately after splitting hiPSCS; 1 μg ml^−1^ of puromycin and 2 μg ml^−1^ of blasticidin were added to E8 flex medium. Cells were kept under antibiotics selection for 10 days. As a readout of successful integration of the transposon cassettes, the RFP signal was checked. All lines or transfected cells expressing light-inducible systems were protected from ambient light with aluminium foil and manipulated for as short a time as possible in a sterile hood. We used a heated surface for cell culture and for checking cells under the microscope whenever possible, as any drop in temperature could induce spurious activation of light-sensitive proteins.

### Organoid differentiation

HiPSCs were cultured in E8 flex medium (Gibco no. A14133-01) with medium replacement every other day until 80% confluency, in the dark. The differentiation protocol was adapted from ref. ^12^. HiPSC colonies were rapidly washed with PBS (Pan Biotech P0436500) and then incubated with accutase (Sigma, catalog no. A6964-100ML) for 4 min at 37 °C. Cells were collected, centrifuged for 3 min at 300*g* and resuspended in E8 flex medium containing 10 μM Y27632 ROCK inhibitor (VWR, catalog no. 688000-5). Cells were counted and plated at a density of 500 cells per well in a 96-well ultra-low attachment U-bottom plate (Corning, catalog no. CLS7007). On the following day, the medium was replaced with fresh N2-B27 medium (50% Neurobasal (Gibco, catalog no. A3582901), 50% DMEM/F12 (Gibco, catalog no. 11320074), 1× N2 (Gibco, catalog no. 17504044), 1× B27 (Gibco, catalog no. 17504044), 1× MEM nonessential amino acids (Sigma, catalog no. M7145-100ML), 1× Glutamax (Gibco, catalog no. 35050038), 0.1 μM β-mercaptoethanol (Merck Millipore, catalog no. 8057400005)) supplemented with 2% Geltrex (Gibco, catalog no. A1413301), 10 μM TGF-β pathway inhibitor (SB431542, Stem Cell Technology, catalog no. 72234, hereon SB43) and 0.1 μM BMP inhibitor (LDN193189, Stem Cell Technology, catalog no. 72147, hereon LDN). Medium was exchanged every 2 days. From day 4 on, organoids were cultured in individual wells of a 24-well plate, ultra-low attachment surface (Corning, catalog no. 3474). Medium was further supplemented with 1 μM retinoic acid (Sigma, catalog no. R2625) until day 8. At day 9, retinoic acid was removed, organoids were further cultured in N2B27 medium supplemented with LDN and SB43 until day 16. For the BMP4-expressing organoids, LDN and SB43 were not supplemented.

### LED board construction and stimulation experiments

For experimental convenience, we decided to build a custom circuit board with 96 blue LEDs that align with the used 96-well plates. To control illumination patterns for each well individually, we opted to wire each LED to a dedicated output line of a constant current LED driver chip (MAX6969). Optimizing for brightness at low supply currents to minimize excess heat, we decided on the Cree XLamp MLESBL with a documented center wavelength at 485 nm and a reported luminous flux of 13.9 lm at 50 mA. We soldered the 96 blue LEDs onto a custom aluminium printed circuit board. The LED printed circuit board serves as a heat sink and is exposed to the incubator environment. A dark PVC hole mask reduces light spill. The assembly is encased in an acrylic frame and the seams sealed with neutrally curing silicone. Two cables leave the case: one to control the shift registers of the driver chips with a microcontroller outside the incubator, and another to power the LEDs (7–9 V) and the logic chips (5 V). We used the serial interface of a microcontroller (Atmel AVR ATmega32) to periodically update the shift registers of the LED drivers according to the desired patterns and to control the output latches. We opted for a control frequency of 1 Hz and specified the illumination patterns with a simple domain-specific language supporting four instructions: turn on the LED for up to 127 s (0x00…0x7E), turn off the LED for up to 127 s (0x80…0xFE), repeat the pattern (0x7F) and halt (0xFF). The code and the schematics for the LED board and the LED drivers are available at https://github.com/BIMSBbioinfo/casled. Stimulations were performed with a 5-s on, 20-s off pattern repeated over the desired time interval (usually 24 h), with the cell culture plate placed directly on top of the LED board. To avoid heating, input voltage was set at 7.6 V for most experiments (below the optimal value for the LEDs used) and temperature of the medium in a lit 96-well plate was checked in a preliminary test with a thermocouple.

### Experimental setup for parallel optogenetic stimulation (DMD setup)

Illumination from a DMD-based projector (DLP LightCrafter 4500, Texas Instruments, modified for on-axis projection by EKB Technologies) was coupled to the rear port of an Observer.Z1 microscope (Zeiss), through a unity magnification relay (×2 AC254-125-A-ML, Thorlabs) with an optical density 2.0 neutral density filter (NE20A-A, Thorlabs). For optical stimulation, illumination from the blue (470 nm) LED of the LightCrafter passed through a GFP filter set (ET-GFP, Chroma) and projected to the sample with a ×10 Plan APO objective. For imaging of RFP, the green (530 nm) LED was used together with a CY3 filter set (ET-CY3/TRITC, Chroma). Projector and/or camera pixel mapping and subsequent control of illumination patterns was performed using the projector plugin for Micromanager v.2.0 gamma^29^. Illumination intensity was controlled using DLP LightCrafter 4500 Control Software (v.3.1.0, Texas Instruments). Emission was detected by a back illuminated sCMOS (PrimeBSI, Teledyne Photometrics). For optogenetic stimulation, samples were illuminated with 470 nm excitation at a power density of 4.7 μW cm^−^^2^ in user defined ROI for 20 s. After stimulation, full FOV RFP images were acquired. This was repeated every minute for 16 h using a custom written Beanshell script in Micromanager. Environmental control during long-term time-lapse imaging was achieved with the Incubator XLmulti S chamber and temperature and CO_2_ controllers (PeCon).

### Laser scanning setup for single-cell stimulations

Scanning-based optogenetic stimulation experiments were conducted using a confocal microscope Leica TCS SP8 (Leica Microsystems) equipped with an environmental (CO_2_ and temperature) control system. Imaging and stimulation were performed using a ×10 Plan APO objective and a white light laser tuned to 488 nm at 1% laser power, within the LAS X (v.3.5.7.23225) software. Scanning-based stimulation of 100 × 100 μm ROI containing a single cell, was performed at 100 Hz unidirectional scan speed. Two sequential scans were performed resulting in 10 s of total exposure. The stimulation protocol was repeated every 30 s for 16–20 h. The scanning-based stimulation setup mimicked the previous LED stimulation pattern, although scanning time was set to 10 s, instead of 5 s of LED illumination, to correct for the off-sample scan time.

For quantifying the NeonGreen signal after single-cell photostimulations (as shown in Extended Data Fig. 2c), we applied a grid of approximately the size of a single cell (10–30 μm side), centered it on the stimulated cell and generated a mask used for ROI selection and quantification, for each grid element, of centroid coordinates and pixel intensity.

### Laser scanning setup for organoid photostimulations

For organoids optogenetic stimulations, the same protocol as before was used to generate embryoid bodies. In the case of hiPSCs carrying the Cre–Lox system, doxycycline was supplemented at day 3 in the N2B27 medium at 1 μg ml^−1^. On the next day, up to four organoids at once were embedded in a drop of cold Geltrex on a glass-bottom dish (WillCo-dish, catalog no. GWSB3522), incubated for 10–15 min at 37 °C and covered with warm N2B27 medium (supplemented with SB43, LDN and retinoic acid). To induce NeonGreen/*SHH* expression in a restricted pole of the organoids, the laser scanning setup was chosen (Leica Sp8 SMD; Supplementary Video 2): a small square ROI of roughly 100–400 μM was selected depending on how many and how far from each other the organoids were positioned, and photostimulated overnight (12–16 h). We found that the minimum laser power (1%) was sufficient for robust activation, while it was necessary to stimulate at low speed (100 Hz) to scan the entire ROI in approximately 10 s (two sequences of roughly 5 s each) and repeat every 30 s. With this regime, the NeonGreen signal appeared already few hours after the initial stimulation (Supplementary Video 2). After the stimulation, the Geltrex was microdissected at a stereomicroscope with two needles, and organoids were retrieved with a pipette and cultured individually in an ultra-low attachment 24-well plate until the desired time point (Corning catalog no. CLS3473). Control organoids, either dox-treated or not, were not induced.

Live imaging of organoids after photostimulation was performed with a Keyence BZ-X710 with GFP and a RFP filters, with ×4 or ×10 magnification, taking 6–10 *z*-stacks 30 μm apart from each other, focused from the bottom to the equator of the organoid.

### RNA extraction with RT–qPCRs

For the experiments shown in Extended Data Figs. 1, 3 and 4, and Supplementary Fig. 2, RNA extraction was performed as follows. Cells were collected by removing medium from 96-well plates, adding 100 μl of home-made Trizol directly onto the cells while keeping the plate on ice, then pipetting up and down a few times and transferring the lysate into a new 1.5 ml tube. Lysates from two or three wells were pooled in each replicate, then RNA was extracted with the Zymo Directzol RNA miniprep kit (Zymo, catalog no. R2051), including DNase I digestion. cDNA was synthesized using 100–200 ng RNA with the Maxima H minus reverse transcriptase (Thermo Fisher, catalog no. EP0751) according to the manufacturer’s protocol and using random hexamers for priming. Here, 5 ng of diluted cDNA were used per qPCR reaction using ROX-supplemented Biozym SYBR green mastermix (Biozym, catalog no. 331416S) and 0.5 μM forward and reverse primers. qPCR reactions were performed in a AB StepOne plus machine under the following cycling conditions: 95 °C for 10 min, then 40 cycles of 95 °C for 15 s and 60 °C for 1 min with fluorescence reading, and a final melting curve step using the StepOne (v.2.3) software.

### Western blotting

The western blot showed in Supplementary Fig. 2d was performed using the Bio-Rad Trans-Blot Turbo Transfer system (semidry blotting apparatus and ready-to-use membranes). Ten micrograms of protein lysates from all samples (HEK293T cells cultured in six-well plates and treated as described in Cell culture, transfections and cell line generation section, lysed with RIPA buffer) were ran for 1.5 h at 150 V on a 4–10% polyacrylamide gel in Tris-glycine buffer. The membrane was cut at roughly 70 kDa according to the ladder (Thermo Fisher, Page Ruler Prestained Plus, catalog no. 26619) and hybridized overnight at 4 °C with anti-GAPDH (Sigma, catalog no. G8795-200UL) and anti-HA (NEB, catalog no. 3724S), both diluted 1:1,000 in 5% milk TBS-T. Membranes were washed three times in TBS-T, hybridized for 1 h with horseradish peroxidase- (HRP-)conjugated secondary antibodies (HRP goat antirabbit, Dako, catalog no. P0448 and HRP goat antimouse, Invitrogen, catalog no. 31430, diluted 1:5,000), washed again and imaged using ECL detection (Thermo Fisher, catalog no. RPN2235) on a Vilber Fusion FX7 Edge imager with the Evolution-CaptEdge Fusion FX Edge (v.18.09) software.

### Flow cytometry

GFP measurements shown in Extended Data Fig. 1 were performed with a BD FACSAriaIII Cell Sorter, with the FACSSiVa (v.8.02) software. HEK293T cells transfected as described in Cell culture, transfections and cell line generation section were collected with 0.05% tripsin, washed twice in PBS and resuspended in 1% bovine serum albumin (BSA) PBS before flow cytometry analysis. The general gating strategy is shown in Extended Data Fig. 1f. A GFP^+^ population was defined to restrict the analysis to transfected cells and thus conservatively compare dark and lit conditions.

### LC–MS proteomics

Cells were transfected and treated as described in the dedicated section. They were then checked by fluorescence after 36 h for RFP knock-down and processed for proteomic analysis as follows. Cells were resuspended in 350 μl of urea buffer (8 M urea, 100 mM Tris-HCl, pH 8.2). Cells were lysed on a Bioruptor sonicator (Diagenode), using ten cycles of sonication (45 s ON, 15 s OFF). Protein concentration was determined by bicinchoninic acid colorimetric assay (Pierce) and a 100 μg aliquot of each protein sample was reduced with 10 mM dithiothreitol for 45 min at 30 °C and alkylated with 100 mM iodoacetamide for 25 min at 25 °C. Proteins were digested using Lys-C (Wako, 1:40, w/w, overnight under gentle shaking at 30 °C) and modified trypsin (Promega, 1:60, w/w, 4 h under rotation at 30 °C). The Lys-C digestion products were diluted four times with 50 mM ammonium bicarbonate before the tryptic digestion, which was stopped through acidification with 5 μl of trifluoroacetic acid (Merck). Next, 15 μg of each resulting peptide mixture were then desalted on Stage Tip^30^, the eluates dried and reconstituted to 15 μl in 0.5% acetic acid. For all the samples, 5 μl were injected on a liquid chromatography with tandem mass spectrometry (LC–MS/MS) system (EASY-nLC 1200 coupled to Q Exactive HF, Thermo), using a 240 min gradient ranging from 2 to 50% of solvent B (80% acetonitrile, 0.1% formic acid; solvent A, 0.1% formic acid in water). Each sample was analyzed in duplicate. For the chromatographic separation 30 cm long capillary (75 μm inner diameter) was packed with 1.9 μm C18 beads (Reprosil-AQ, Dr. Maisch high-performance liquid chromatography). On one end of the capillary nanospray tip was generated using a laser puller, allowing fretless packing (P-2000 Laser Based Micropipette Puller, Sutter Instruments). The nanospray source was operated with a spray voltage of 2.0 kV and an ion transfer tube temperature of 260 °C. Data were acquired in data dependent mode, with one survey MS scan in the Orbitrap mass analyzer (120,000 resolution at 200 *m/z*) followed by up to ten MS/MS scans (30,000 resolution at 200 *m/z*) on the most intense ions. Normalized collision energy was set to 26. Once selected for fragmentation, ions were excluded from further selection for 30 s, to increase new sequencing events. Raw data from proteomics data processing and analysis were analyzed using the MaxQuant proteomics pipeline (v.1.6.10.43) and the built in the Andromeda search engine^31^ with the UniProt Human database. Carbamidomethylation of cysteines was chosen as fixed modification, oxidation of methionine and acetylation of N terminus were chosen as variable modifications. The search engine peptide assignments were filtered at 1% FDR and the feature match between runs was enabled. For protein quantification, label-free quantitation (LFQ) intensities calculated by MaxQuant were used^32^. The minimum LFQ ratio count was set to 2 and a MS/MS spectrum was always required for LFQ comparison of the precursor ion intensities. Data quality was inspected using the in-house developed tool PTXQC^33^. After removing reverse and contaminants hits, LFQ intensities were log_2_ transformed and proteins with less than four valid values in each condition were filtered out. Proteins with differential expression between conditions were tested with Student’s *t*-test with Benjamini–Hochberg FDR set at 0.05. Processed data are available in the Supplementary Tables 1 and 2.

### Plasmids

Supplementary Table 3 contains the name, description and information on availability of the plasmids used in this study.

### Bulk RNA-seq

Cells were treated exactly as for the proteomics experiment and as described in the dedicated section, in two additional replicates per condition. RNA was extracted with home-made Trizol by organic phase separation and RNA precipitation. Total RNA-seq libraries were performed as follows: 1 μg of total RNA per sample was first depleted of ribosomal RNA using the RiboCop ribosomal RNA Depletion Kit (Lexogen, catalog no. 144) according to the manufacturer’s instructions. The rRNA-depleted samples were then processed with the TruSeq mRNA stranded kit from Illumina. Libraries were then sequenced on a NextSeq with 1 × 76 cycles. Fastq data were generated with the bcl2fastq program and fed to the PiGx analysis pipeline^34^, which was used with default settings with a custom reference GRCh38 human genome supplemented with two extra chromosomes carrying the CasRx–T2A-GFP cassette and the TagRFP cassette, and a custom annotation made of the Gencode v34 human annotation supplemented with two extra entries for the CasRx–T2A-GFP and the TagRFP genes. For further analyses, we used the STAR/Deseq2 PiGx output.

### Live-cell imaging for GFP and RFP quantification in HEK cells

After 6, 12, 25 or 50 h of light induction or the respective dark controls, images for GFP and RFP were acquired on an inverted Nikon Ti-E microscope with a ×4 1.4-numerical aperture objective and Andor iXON Ultra DU-888 camera; *Z*-stacks had 1.5 µm spacing over a 40 µm range. GFP had 300 ms of exposure; Sola 50% on 6–12 h, 12% on 25–50 h. RFP had 100 ms of exposure; Sola 20%. All these images were taken with live cells in black 96-well plates. *Z*-stacks were used for maximum intensity projection within ImageJ, and the projection were used for signal quantification with a macro running the ImageJ Subtract Background plugin with a rolling ball radius of 50, and then the Measure function for signal intensity. This quantification assumes that all wells contain on average the same number of cells, which were seeded in the beginning of the experiment. For some of the wells, we noticed a pipetting artifact on a side, producing an area devoid of cells. We manually selected a ROI that excluded this area for all wells, and we applied this before running the signal measurement macro. This experiment was performed blindly: I.L. transfected the cells and performed the light stimulation, C.C.J. performed the imaging without knowing or seeing the sample labels, then I.L. ran the quantification and then reassigned the original labels to the well names.

### Immunofluorescence of hiPSCs and organoids

HiPSCs or organoids were rapidly washed in cold PBS and fixed in 4% PFA for 10 min in a multiwell plate with agitation. For whole-mount imaging, permeabilization and blocking were performed for 1 h at room temperature in PBS solution with 0.1% Triton-X, 0.2% BSA and 4% normal donkey serum. Organoids were incubated with primary antibodies overnight at 4 °C in blocking solution (PBS supplemented with 0.2% BSA and 4% normal donkey serum). The following primary antibodies were used in immunostaining: Anti-FOXA2 (R&D systems, catalog no. AF2400; 1:100), Anti-OLIG2 (Sigma, catalog no. HPA003254-100UL; 1:1,000), Anti-NKX6.1 (Sigma, catalog no. HPA036774-100UL; 1:500), anti-mNeonGreen (Fisher Scientific, catalog no. 32F6; 1:50), anti-Hb9 (DSHB, catalog no. 81.5C10; 1:100), anti-Isl1/2 (DSHB, catalog no. 39.4D5; 1:100), anti-CHX10 (Novus Biologicals, catalog no. NBP1-85576, 1:100); anti-PDGFRB (Cell Signaling Technology, catalog no. 3169; 1:200). On the following day, hiPSCs and organoids were washed three times for 10 min, with agitation and with washing solution (PBS supplemented with 0.1% Triton-X, 0.2% BSA). Secondary antibodies and 4,6-diamidino-2-phenylindole (DAPI) (Sigma, catalog no. D9542) were then incubated at room temperature for 1 h in blocking solution. The following secondary antibodies were used at 1:1,000 dilution in blocking solution: Alexa Fluor 647 antiRabbit (Thermo Fisher, catalog no. A21244), Alexa Fluor 647 anti-Goat (Thermo Fisher, catalog no. A21447), depending on the primary antibody. Samples were then washed again three times for 10 min, with agitation, in washing solution. For mounting, the organoids were placed in the center of a slide, washing solution was carefully removed and one drop of Prolong Gold antifade reagent (Thermo Fisher, catalog no. P36930) was placed on top of each organoid. A coverslip was placed on top and the slides were allowed to dry at room temperature overnight in the dark. For hiPSCs, the mounting media was added directly in the cell culture plates where cells were seeded (Thermo Fisher, catalog no. P10144).

For organoid cryo-sections: after fixation, organoids were allowed to settle in 1 ml of 40% sucrose solution overnight at 4 °C. On the following day, they were embedded in 13%:10% gelatin:sucrose solution and positioned inside an embedding mold (Sakura catalog no. 4566), rapidly moved to dry ice to freeze and then placed at −80 °C for storage. Blocks were removed from −80 °C and allowed to warm inside the cryostat to sectioning temperature (−20 °C) for 15 min. Sectioning was performed using a cryostat (Thermo Fisher Cryostar NX70) and set to produce 10-μm-thick sections. Cut sections were collected on slides (Thermo Fisher, catalog no. J1800AMNZ) and stored long term at −80 °C. To perform immunostaining, slides were allowed to warm to room temperature for 10 min, incubated for 5 min with 37 °C PBS to remove embedding solution. Permeabilization and blocking, as well as incubation with primary and secondary antibodies, were done as described above for whole-mount organoids. Images were acquired using a confocal microscope (Leica TCS SP8) using a ×10 dry or a ×20 glycerol immersion objective. *Z*-stacks and final images were processed using Fiji-ImageJ, to produce *z* projections, subtract background and adjust minimum and maximum values for better image visibility. For FOXA2 stainings in Extended Data Fig. 4d, we used the ImageJ outlier removal function to remove oversaturated pixels that probably resulted from antibody precipitation.

### Spatial transcriptomics: Visium experiments and analysis

PET membranes (Millipore Millicell Hanging Cell Culture Insert, PET 3 μm, 24-well, catalog no. MCSP24H48) were positioned in glass-bottom black 24-well plate (Greiner Bio-one, catalog no. 662892), after cutting away the plastic holders, hence making the membrane touch the bottom of the well with no gaps in-between (this step was performed to ensure no light scattering or diffusion). Circular black photomasks were sticked underneath the bottom of the plate. Membranes were coated with 100 μl of cold Geltrex. iPSCs were splitted to single cells, as described above, and 275,000 cells resuspended in 100 μl were cultured on coated membranes generating a stable monolayer. Additional warm E8 media (300 μl) was pipetted around the plastic scaffold. Plates were incubated at 37 °C for 2 or 3 h until cells attachment. Samples were prepared in duplicates with the intent to perform control quantifications for each of those before the final Visium experiment. Plates were kept wrapped in aluminium foil to avoid light exposure. Before starting with the first 24 h of light induction, media was changed to one-half E8 flex, one-half COM1 and 1 μg ml^−1^ of doxycycline.

The plate with the cells to be induced was covered on top by a black velvet lid and positioned onto the blue LED plate. The control sample (0 h) was kept in the dark during the whole time course. Media was changed to one-quarter E8 flex and three-quarters COM1 between 24 and 48 h of induction. Once the time course finished, the four samples were transferred to a Visium Spatial Gene Expression slide (10x Genomics) as follows: the plastic structure that surrounds the membrane was carefully held with tweezers and turned upside down to get rid of the media; membranes were delicately washed twice with 100 μl of PBS and, by using a scalpel, delicately isolated from the plastic device. By using tweezers, the membranes were then slowly stuck onto a Visium Spatial Gene Expression slide with cells facing on to it. The more the membrane was kept flat, the more efficient the cells transferred. The Visium Spatial Gene Expression protocol was followed, according to the manufacturer’s instructions (10x Genomics).

Fastq files were first processed for retrieving transcript counts with positional information with the spaceranger software (10c Genomics, v.1.2.0). The output of spaceranger was loaded into Seurat (v.4.0) within RStudio with R v.4.0.4, and each sample was subsetted into seven concentric circles with the center being set according to the stimulation pattern observed by fluorescent microscopy (and after checking that different radii would yield stable results in the samples with SHH induction, subsetting from five to ten concentric circles and finally settling for an intermediate size). The central spots selected for each sample from 0 to 120 h had the following barcodes: CACATGATTCAGCAAC, CAATTTCGTATAAGGG, CAATTTCGTATAAGGG and GGAGGGCTTGGTTGGC (the last was north-west from the physical center as the induction was not centered). At this point, concentric circles were drawn by taking all spots with a distance from the center less than 500, 775, 1,050, 1,325, 1,600, 1,825 and more than 1,825 for the c1–c7 areas. Within these subsets of spots, the transcript counts for a *SHH* gene set comprising *SHH*, *NKX6-1*, *NKX6-2*, *NKX2-2*, *NKX2-1*, *FOXA2*, *FOXG1* and *OLIG2* were added and normalized for the total transcript counts of each subset, and then further normalized by the mean of the counts for all spatial subsets c1–c7. As controls, we either randomized the genes in the gene set 1,000 times, or the center spot 1,000 times, and then computed an exact *P* value for each subset gene set enrichment testing the hypothesis of the enrichment being larger than the random control. The signal was stable with varying binning sizes (from six to nine) and over cumulative analysis per single capture spot. We carried out same analysis for a gene set composed of HSF1 targets to check for heat-related effects (from the msigdb hallmark gene set *hsf1 master regulator of heat shock response*).

### Spatial transcriptomics: molecular cartography experiments and analysis

Control and SHH-induced organoids at 12 days postinduction were washed twice in PBS, submerged for 2 h in PaxGene fixation reagent at room temperature (Qiagen, catalog no. 765312), kept overnight at 4 °C in PaxGene stabilization reagent, then soaked in a 40% m:v sucrose:PBS solution for 30 min, optimal cutting temperature-embedded, snap-frozen and cut in 10-μm-thick cryo-sections that were placed directly on a glass slide provided by Resolve Biosciences for molecular cartography analysis. Slides were shipped to Resolve Biosciences, where they were processed for multiplexed single-molecule fluorescent in situ hybridization of a panel of 88 transcripts of interest, including those showed in Fig. 3 and Extended Data Fig. 3 and additional neuronal markers and housekeepers. Before this experiment, we optimized the fixation and embedding conditions to ensure optimal cryo-sectioning and the compatibility of our sections with the Resolve Biosciences imaging setup, given the background fluorescence of our organoids expressing RFP and NeonGreen. We concluded that PaxGene fixation ensured better performance over 4% PFA fixation and fresh-frozen samples for the background fluorescence, while optimal cutting temperature embedding was preferable over gelatin embedding.

Images and transcript quantification data provided by Resolve were processed using Fiji-imageJ and the Polylux v.1.9.0 plugin for transcripts visualization over a binarized gray mask of the processed organoids (Fig. 3h and Extended Data Fig. 3d), or imported in R and processed with Seurat v.4.0.5 on R v.4.0 for performing log-normalization, producing the heatmap in Extended Data Fig. 3c with the DoMultiBarHeatmap() package (https://github.com/elliefewings/DoMultiBarHeatmap), and the dimensionality reduction plots (uniform manifold approximation and projection (UMAP) performed on 12 PCs) in Extended Data Fig. 3d.

For computing the distance distribution shown in Fig. 3i, the cell segmentation ROI provided by Resolve were imported in ImageJ where they were used for computing their centroid coordinates. The distance of the centroids of cells with at least five counts of each transcript of interest was computed from that of each cell with at least five *SHH* counts, and only the distances with the nearest SHH^+^ cell were kept for further analysis.

### scRNA-seq

Organoids were dissociated using accutase, followed by washing with growth medium and filtration through a 40 μm cell strainer. Cells were then pelleted and resuspended in PBS supplemented with 0.01% BSA. Cells were counted with a hemocytometer and diluted to a suspension of roughly 300 cells per μl. Cells were encapsulated together with barcoded microparticles (Macosko- 2011-10V+, ChemGenes) using the Dolomite Bio Nadia instrument and using the standard manufacturer’s dropseq-based scRNA-seq protocol. Droplets were broken immediately after collection and cDNA libraries generated as previously described^35^: first, strand cDNA was amplified by equally distributing beads from one run to 24 Smart PCR reactions (50 μl volume; 4 + 9 to 11 cycles); 20 μl of fractions of each PCR reaction were pooled, then double-purified with 0.6 volumes of AMPure XP beads. Amplified cDNA libraries were assessed and quantified on a BioAnalyzer High Sensitivity Chip (Agilent) and the Qubit double-stranded DNA HS Assay. Then, 900 pg of each cDNA library was fragmented, amplified (13 cycles) and indexed for sequencing with the Nextera XT v2 DNA sample preparation kit (Illumina) using custom primers enabling 3′-targeted amplification. The libraries were purified with AMPure XP beads, quantified and sequenced on Illumina sequencers (first run, concentration 2 pM; Hiseq 3000/4000 SBS kit (150 cycles) paired-end mode; read 1 = 75 using the custom primer Read1CustSeqB (8 bp) read 2 = 75. Second run: concentration 2 pM; NextSeq 500/550 High Output v.2 kit (75 cycles) in paired-end mode; read 1 = 21 bp using the custom primer Read1CustSeqB (8 bp) read 2 = 63 bp).

Data were produced and demultiplexed by the BIMSB genomics platform, and fastq files were used as input to the Spacemake pipeline^36^ (https://github.com/rajewsky-lab/spacemake), used with default parameters in scRNA-seq run mode and with a minimum of 500 UMI and maximum of 10,000 cells cutoffs, to generate a gene expression matrix for each of the fastq file. Since two sequencing runs were performed for each sample, Spacemake was used in merge mode to create a merged gene expression matrix for each of the four samples. Such matrices were then used as input for Seurat v.4.0.5 on R v.4.0 to create a merged Seurat object. The Seurat object was filtered for cells with more than 800 UMIs and less than 5% mitochondrial RNA read content, data were then log-normalized, scaled and used for principal component analysis dimensionality reduction on 2,000 variable features. Ten PCs were used for subsequent clustering and UMAP (Extended Data Fig. 4a). Clustering with a 0.4 resolution identified eight clusters: 0 and 2, composed of mainly *SHH*-induced samples, shared similar gene expression patterns and differed mainly in the number of UMIs and housekeeping transcripts content (Extended Data Fig. 4b), and were marked by neural progenitor markers, so they were annotated as *SHH* progenitors; 1 and 3 were similarly composed of control progenitors; 4 was marked by neuronal marker genes; 5 was mainly composed of low-UMI cells with enriched ribosomal protein genes expression and was therefore annotated as low-quality and excluded; 6 was marked by extracellular matrix components and labeled as pericyte-like cells; 7 was composed of very few cells with extremely high UMI content and enriched nuclear and/or noncoding RNA markers, and removed for further analyses. After this second filtering, principal component analysis and UMAP analyses were performed again on the subset to produce the plots shown in Fig. 4 and Extended Data Fig. 4.

To ensure that the two progenitor populations (CTR and SHH) were not artifactual, we also applied data integration using the Seurat integration workflow (Extended Data Fig. 7a,b). The cluster structure for neuronal progenitors remains relatively stable after integration of control and induced datasets. More precisely, 86 and 66% cells from the two control progenitor clusters ending up in two integrated control progenitor clusters, and 85 and 77% cells from the two SHH progenitors clusters ending up in two integrated SHH progenitor clusters. The rest of the cells ended up in a few additional clusters (mainly cluster 4) that contained both control and progenitor cells, but represented roughly only 10% of all progenitors. De novo clustering each sample individually instead resulted in similar cell populations, in which most cells (progenitor cells) end up in 3–4 clusters mainly characterized by different UMI counts as in the merged object, a neuronal cluster and one or two clusters with the pericyte-like signature found only in the SHH-induced organoids.

Gene set enrichment analysis on gene ontology terms and Kyoto Encyclopedia of Genes and Genomes (KEGG) pathways was performed with the functions gseGO() and gseKEGG() in clusterProfiler v.3.18.1 (ref. ^37^), with a minGSSsize set to ten, maximum set to 500 and pvalueCutoff set at 0.05. Input to this analysis was a log fold-change-ranked list of differentially expressed genes computed with the FindMarkers() function in Seurat with min.pct and logfc.threshold both set at 0.1, between control and SHH-induced cells within the progenitor clusters 0–3.

Module scores shown in Fig. 4a,d were computed with the AddModuleScore function in Seurat, with the following genes: *SHH*, *FOXA2*, *NKX2-2*, *OLIG2*, *NKX6-1*, *NKX6-2*, *PTCH1*, *HHIP* in the SHH module, *FOXA2*, *NKX6-1*, *SHH*, *FERDL3L*, *ARX, LMX1B* in the floor plate, *NKX6-1*, *NKX2-2*, *NKX2-9* in the p3, *SP8*, *NKX6-1*, *OLIG2* in the pMN, *IRX3*, *IRX5*, *PAX6*, *DBX2*, *DBX1*, *SP8*, *NKX6-2*, *PRDM12*, *NKX6-1*, *FOXN4* in the p0-2, *MSX2*, *PAX3*, *OLIG3*, *IRX3*, *IRX5*, *PAX6*, *PAX7*, *GSX2*, *ASCL1*, *GSX1*, *GBX2*, *DBX2*, *DBX1*, *SP8* in the dp1-6, *LMX1A*, *MSX1*, *MSX2*, *PAX3*, *WNT1* in the roof plate modules (selected from ref. ^16^).

Spatial reconstruction shown in Fig. 4e–i was performed with NovoSpaRc^18^ (https://github.com/rajewsky-lab/novosparc), with alpha set to 0.5. Inputs were: a gene expression matrix composed of cells in clusters 0–3, additionally filtered for having more than 1,200 UMIs, a *z*-score scaled expression matrix of positional marker genes selected as follows and a list of 1,000 highly variable genes computed in Seurat from the clusters 0–3 and integrated with the 32 positional marker genes. Gene expression data and annotation metadata from a developing spinal cord atlas were downloaded from ref. ^16^, and filtered for cells annotated in one of the 13DV progenitor domains. The FindMarkers() function was used to compute marker genes for each domain, and an a priori set of known positional markers was complemented with the identified markers with highest fold-change, filtered for being expressed in the organoids data (the final list is shown in Fig. 4e). To control for the robustness of the reconstruction performed on all cells, we sampled 100 × 4,000–5,000 random cells and found highly similar results on the marker genes. To compare the novoSpaRc reconstruction of organoids data with mouse developing spinal cord data, we generated a *z*-score scaled gene expression matrix for 1,000 variable genes from the novoSpaRc data and computed a correlation matrix with the *z*-score scaled mouse data after filtering for genes present in both datasets, converting from human to mouse with an orthology table obtained from Biomart and additionally filtered for unambiguous orthology. The same procedure was then repeated, with the same positional marker genes, for spatial reconstruction of organoids data based on a human neural tube scRNA-seq expression matrix^17^.

### Whole-mount organoids in situ hybridization

Organoids were collected in PBS and fixed with 4% PFA for 20–30 min, washed with PBST (PBS with 0.1% Tween) and rehydrated through the series of increased methanol concentrations in PBST (25%/50%/75%/100%). Organoids were stored at −80 °C in 100% methanol. Before hybridization, organoids were rehydrated through a series of decreased methanol concentrations in PBST (75%/50%/25%) and washed twice with PBST at room temperature, followed by Proteinase K treatment (5 μg ml^−1^, Roche) for 2 min, two PBST washes and refixation with 4% PFA for additional 10 min at room temperature. After two PBST washes, organoids were prehybridized with prewarmed prehybridization buffer (5× SSC, 50 mM dithiothreitol, 1 mM EDTA, 1× Denhardt’s solution, 100 μg ml^−1^ transfer RNA, 50% formamide, 0.1% Tween), at 65 °C for 2 h. Prehybridization solution was replaced with hybridization solution containing 0.5 μg of in vitro transcribed DIG-labeled RNA probe and hybridization was performed overnight at 65 °C. After hybridization, organoids were washed with 1:1 prehybridization buffer 2× SSC prewarmed to the hybridization temperature for 1 × 15 min, 2× SSCT buffer prewarmed to the hybridization temperature for 3 × 15 min, twice with PBST at room temperature, followed by incubation for 2 h with blocking solution (PBST with 5% normal goat serum) and overnight with anti-DIG antibody (Roche catalog no. 11333089001, 1:2,000 dilution). Organoids were washed 3× with PBST, 2× with NTMT buffer (50 ml NTMT: 1 ml 5 M NaCl, 2.5 ml 2 M Tris-HCl pH 9.5, 2.5 ml 1 M MgCl_2_, 5 ml 10% Tween20, water up to 50 ml). For colorimetric detection, organoids were stained with NBT/BCIP solution (Roche) until a clear signal was observed, followed by washing with PBST and refixation with 4%PFA.

### Primers and guide RNA sequences

All primer and guide RNA sequences are provided in Supplementary Note 2.

### Statistical analyses

For imaging and qPCR measurements we generally adopted a parametric approach by first applying exploratory analysis of variance analysis to each experimental variable (for example, time, light, sgRNA or dox and promoter where applicable), followed by *t*-test and Benjamini–Hochberg correction on the indicated comparisons. For other measurements (for example, nonnormally distributed variables or measurements with a high number of single observations), we used Wilcoxon–Mann–Whitney tests and the Benjamini–Hochberg correction. Finally, for specific cases we computed exact *P* values via random permutations as described, for example, for the Visium data. All relevant and significant corrected *P* values are displayed in the figures.

### Reporting summary

Further information on research design is available in the Nature Portfolio Reporting Summary linked to this article.

## Online content

Any methods, additional references, Nature Portfolio reporting summaries, source data, extended data, supplementary information, acknowledgements, peer review information; details of author contributions and competing interests; and statements of data and code availability are available at 10.1038/s41592-023-01986-w.

### Supplementary information

Supplementary Information Supplementary Notes 1 and 2 (list of primers and guide RNAs), Figs. 1–3 and references.

Reporting Summary

Supplementary Table 1 Proteomic (LFQ and iBAQ values for three biological replicates times two technical replicates per condition along with annotations and statistics; conditions are nontargeting guide RNA, NT and RFP-targeting guide RNA, *RFP* or *PS18*; see Methods for more details on the analysis). *P* values were computed with two-sided Benjamini–Hochberg-corrected *t*-test.

Supplementary Table 2 Transcriptomic data (DeSeq2 output from the PiGx pipeline for two biological replicates per condition; see Methods for more details on the analysis) for HEK293T cells where RFP was knocked down with CasRx. *P* values were computed with Deseq2.

Supplementary Table 3 List of the plasmids used in this work.

Supplementary Video 1 SHH induction in hiPSCs: a laser scanning setup was used to stimulate SHH expression with the PA-Cre–Lox system^5^ in a squared ROI at the center of a larger FOV. On the left, the RFP and GFP channels for the entire FOV at the end of induction; on the right, the photostimulated ROI in the GFP channel. Stimulation was performed overnight (roughly 16 h).

Supplementary Video 2 SHH induction in neural organoids: induction performed as in video S1. Four embryoid bodies are arranged in a dish and embedded in matrigel to use a single ROI to stimulate a limited region of all of them at once. On the left, the RFP and GFP channels for the entire FOV at the end of induction; on the right, the photostimulated ROI in the GFP channel. Stimulation was performed overnight (roughly 18 h).

### Source data

Source Data Fig. 1 Data points for Fig. 1d,e.

Source Data Fig. 2 Data points for Fig. 2d,f and Extended Data Fig. 2c.

Source Data Extended Data Fig. 1 Data points for the Extended Data Fig. 1a–e.

Source Data Extended Data Fig. 3 Data points for Extended Data Fig. 3b,d–g.

Source Data Extended Data Fig. 4 Data points for Extended Data Fig. 4c,e,h.

## Data Availability

All raw and processed sequencing data have been deposited in the Gene Expression Omnibus under the accession no. GSE185022. Molecular Cartography data have been deposited on Zenodo^38^ (10.5281/zenodo.6143560) under the accession no. 6143561. All other datasets are provided as Source data files that are available with this paper.

## Code availability

All analyses were performed with publicly available or commercial software as indicated in Methods and in the Reporting Summary. Additional software developed in the Rajewsky laboratory used in this work is available on GitHub: https://github.com/rajewsky-lab/spacemake; https://github.com/rajewsky-lab/novosparc and https://github.com/BIMSBbioinfo/casled. Additional scripts describing the analyses performed here have been deposited on Zenodo^38^ (10.5281/zenodo.6143560).

## References

[1] Lancaster MA (2013). Cerebral organoids model human brain development and microcephaly. Nature.

[2] Kelley KW, Pașca SP (2022). Human brain organogenesis: toward a cellular understanding of development and disease. Cell.

[3] Nihongaki Y (2017). CRISPR-Cas9-based photoactivatable transcription systems to induce neuronal differentiation. Nat. Methods.

[4] Yamada M, Suzuki Y, Nagasaki SC, Okuno H, Imayoshi I (2018). Light control of the Tet gene expression system in mammalian cells. Cell Rep..

[5] De Santis R, Etoc F, Rosado-Olivieri EA, Brivanlou AH (2021). Self-organization of human dorsal-ventral forebrain structures by light induced SHH. Nat. Commun..

[6] Abudayyeh OO (2017). RNA targeting with CRISPR-Cas13. Nature.

[7] Cox DBT (2017). RNA editing with CRISPR-Cas13. Science.

[8] Konermann S (2018). Transcriptome engineering with RNA-targeting type VI-D CRISPR effectors. Cell.

[9] Ribes V, Briscoe J (2009). Establishing and interpreting graded Sonic Hedgehog signaling during vertebrate neural tube patterning: the role of negative feedback. Cold Spring Harb. Perspect. Biol..

[10] Nihongaki Y, Yamamoto S, Kawano F, Suzuki H, Sato M (2015). CRISPR-Cas9-based photoactivatable transcription system. Chem. Biol..

[11] Loew R, Heinz N, Hampf M, Bujard H, Gossen M (2010). Improved Tet-responsive promoters with minimized background expression. BMC Biotechnol..

[12] Zheng Y (2019). Dorsal-ventral patterned neural cyst from human pluripotent stem cells in a neurogenic niche. Sci. Adv..

[13] Marques S (2018). Transcriptional convergence of oligodendrocyte lineage progenitors during developmentd. Dev. Cell..

[14] Chamling X (2021). Single-cell transcriptomic reveals molecular diversity and developmental heterogeneity of human stem cell-derived oligodendrocyte lineage cells. Nat. Commun..

[15] Philippidou P, Dasen JS (2013). Hox genes: choreographers in neural development, architects of circuit organization. Neuron.

[16] Delile J (2019). Single cell transcriptomics reveals spatial and temporal dynamics of gene expression in the developing mouse spinal cord. Development.

[17] Rayon T, Maizels RJ, Barrington C, Briscoe J (2021). Single-cell transcriptome profiling of the human developing spinal cord reveals a conserved genetic programme with human-specific features. Development.

[18] Nitzan M, Karaiskos N, Friedman N, Rajewsky N (2019). Gene expression cartography. Nature.

[19] Comer JD, Alvarez S, Butler SJ, Kaltschmidt JA (2019). Commissural axon guidance in the developing spinal cord: from Cajal to the present day. Neural Dev..

[20] Moses L, Pachter L (2022). Museum of spatial transcriptomics. Nat. Methods.

[21] Kutejova E, Sasai N, Shah A, Gouti M, Briscoe J (2016). Neural progenitors adopt specific identities by directly repressing all alternative progenitor transcriptional programs. Dev. Cell..

[22] Ogura T, Sakaguchi H, Miyamoto S, Takahashi J (2018). Three-dimensional induction of dorsal, intermediate and ventral spinal cord tissues from human pluripotent stem cells. Development.

[23] Li P (2018). Morphogen gradient reconstitution reveals Hedgehog pathway design principles. Science.

[24] Cederquist GY (2019). Specification of positional identity in forebrain organoids. Nat. Biotechnol..

[25] Xu H, Jiao D, Liu A, Wu K (2022). Tumor organoids: applications in cancer modeling and potentials in precision medicine. J. Hematol. Oncol..

[26] Rogers KW, ElGamacy M, Jordan BM, Müller P (2020). Optogenetic investigation of BMP target gene expression diversity. eLife.

[27] Wang X (2018). Genome-wide analysis of PDX1 target genes in human pancreatic progenitors. Mol. Metab..

[28] Fogarty MP, Emmenegger BA, Grasfeder LL, Oliver TG, Wechsler-Reya RJ (2007). Fibroblast growth factor blocks Sonic Hedgehog signaling in neuronal precursors and tumor cells. Proc. Natl Acad. Sci. USA.

[29] Edelstein AD (2014). Advanced methods of microscope control using *μ* Manager software. J. Biol. Methods.

[30] Rappsilber J, Mann M, Ishihama Y (2007). Protocol for micro-purification, enrichment, pre-fractionation and storage of peptides for proteomics using StageTips. Nat. Protoc..

[31] Cox J (2011). Andromeda: a peptide search engine integrated into the MaxQuant environment. J. Proteome Res..

[32] Cox J (2014). Accurate proteome-wide label-free quantification by delayed normalization and maximal peptide ratio extraction, termed MaxLFQ. Mol. Cell Proteom..

[33] Bielow C, Mastrobuoni G, Kempa S (2016). Proteomics quality control: quality control software for MaxQuant results. J. Proteome Res..

[34] Wurmus R (2018). PiGx: reproducible genomics analysis pipelines with GNU Guix. Gigascience.

[35] Wyler E (2021). Transcriptomic profiling of SARS-CoV-2 infected human cell lines identifies HSP90 as target for COVID-19 therapy. iScience.

[36] Sztanka-Toth TR, Jens M, Karaiskos N, Rajewsky N (2022). Spacemake: processing and analysis of large-scale spatial transcriptomics data. Gigascience.

[37] Yu G, Wang LG, Han Y, He QY (2012). clusterProfiler: an R package for comparing biological themes among gene clusters. OMICS.

[38] Legnini, I. et al. Spatio-temporal, optogenetic control of gene expression in organoids. *Zenodo*10.5281/zenodo.6143560 (2022).

