# Supplementary material for: Spatiotemporal, optogenetic control of gene expression in organoids
Source: Nat Methods. 2023 Sep 21;20(10):1544–52. doi: 10.1038/s41592-023-01986-w (PMC10555836; doi:10.1038/s41592-023-01986-w)
Supplement: Supplementary file 1 — Supplementary Notes 1 and 2 (list of primers and guide RNAs), Figs. 1–3 and references. [file 41592_2023_1986_MOESM1_ESM.pdf]

nature methods

Article

https://doi.org/10.1038/s41592-023-01986-w

Spatiotemporal, optogenetic control of gene expression in organoids

In the format provided by the authors and unedited

nature methods

Article https://doi.org/10.1038/s41592-023-01986-w

Spatiotemporal, optogenetic control of gene expression in organoids

In the format provided by the authors and unedited

Supplementary note: light-inducible knock-down of reporter and endogenous transcripts.

Introduction

While gene activation based on light-inducible transcription (Polstein et al., 2015, Nihongaki et al., 2015a, Nihongaki et al., 2017, De Santis et al., 2021) has been effectively used in cell culture and even in organotypic culture and *in vivo* (Yamada et al., 2018), spatio-temporal control of RNA knock-down poses several unmet challenges.

Previous efforts in this direction combined light-responsive protein modules (Kennedy et al., 2010, Renicke et al., 2013, Kawano et al., 2015) with CRISPR/Cas9 (Nihongaki et al., 2015b, Zhou et al., 2017), to induce irreversible genetic mutations. However, the results can be difficult to interpret due to mutational heterogeneity and limited efficacy. An alternative approach, based on light-inducible knock-downs, has only been reported in cell lines, for example with photo-caged oligonucleotides/siRNAs (e.g. Mikat et al., 2007), or more recently with a genetically encoded optogenetic RNA interference (RNAi) system (Pilsl et al., 2020). While these methods are efficient and allow for precise temporal control in cell lines, their efficacy is unknown in more complex tissues and may be limited. For example, the RNAi approach used in Pilsl et al., is constitutively active in the dark and is inactivated by photo-stimulation, making it challenging to spatially control the knock-down of a given target in an organoid or *in vivo*. Furthermore, RNAi in general is known to suffer from off-target effects.

We therefore explored two alternative strategies for achieving optogenetic activation of CRISPR/Cas13, a relatively new molecular tool for efficient and programmable RNA targeting, by either controlling its transcription with photo-stimulation, or its enzymatic activity.

RNA knock-down by light inducible transcription of CRISPR/CasRx

We first tested the efficacy of constitutively expressed or light-inducible CasRx in knocking down reporter and endogenous transcripts (Fig. S2a) and here report our results. Constitutively expressed CasRx was able to efficiently knock-down a Tet-ON RFP reporter with a single guide RNA (PS18 adopted from Abudayyeh et al., 2016; hereafter referred to as “RFP guide RNA”; Fig. S2b). However, GFP (which tags the CasRx cassette) was also depleted for reasons that we do not fully understand (Fig. S2c). This effect was not restricted

to GFP only, but also CasRx was depleted when targeting RFP (Fig. S2d), suggesting that such cross-talk occurs at the RNA level, as CasRx and GFP are co-expressed within the same cistron but GFP is cleaved off CasRx via a self-cleaving peptide. GFP depletion upon RFP targeting seems an effect restricted to these two genes, since targeting a luciferase mRNA carrying the same target sequence with the same guide RNA did not induce any GFP nor CasRx depletion and the guide RNA targeting RFP did not induce any GFP nor CasRx knock-down in absence of RFP (Fig. S2d). On the other hand, exchanging the GFP tag with a different fluorescent protein (UnaG) retained this effect (Fig. S2e). However, this off-targeting seemed limited to the overexpressed cassette. In fact, to examine the occurrence of global off-targeting effects, which have been recently reported in certain conditions (Ai et al., 2022, Shi et al., 2021), we performed genome-wide mass-spectrometry-based quantitative proteomics in three biological and two technical replicates per condition. The only proteins with a statistically significant fold change greater than 2 when comparing cells transfected with a non-targeting guide (NT) vs. an RFP-targeting guide were RFP, CasRx and GFP (Fig. S2f). We also sequenced total RNA in two replicates per condition and found only six differentially expressed transcripts: four were highly homologous to 18S ribosomal RNA (a typical artifact of ribodepletion) and the remaining two were RFP and CasRx-GFP, with only RFP having a fold-change larger than 2 (Fig. S2g). Therefore, genome-wide protein and RNA quantification demonstrated the knock-down specificity in the tested conditions and within the analyzed time frame, with the exception of the CasRx-GFP mRNA.

Given the targeting efficacy and specificity of the constitutive CasRx, we tested whether a light-induced CasRx is also capable of knocking down RFP. We transfected HEK cells with the SCPTS CasRx systems together with either a non-targeting or an RFP-targeting guide and observed RFP knock-down efficiencies of 40-60% in the induced (lit) state, with residual activity of 10-30% in the uninduced (dark) state, depending on the construct (Fig. S2h). As before, GFP was unexpectedly depleted when CasRx was programmed to target RFP (Fig. S2i). We then tested the CaSP2-CasRx system, which provided a good trade-off between targeting efficacy and leakage, on endogenous targets. We designed two guide RNAs complementary to the circular RNA CDR1as and adopted a previously published sequence for the STAT3 mRNA (Konermann et al., 2018) and validated them with a constitutive CasRx (Fig. S2j). We transfected one CDR1as guide and the STAT3 guide together with the SCPTS-CaSP2-CasRx system, stimulated the cells with blue light for 24-36 hours and

performed qRT-PCR for target quantification. As shown in Fig. S2k, we achieved ~71% and ~45% knock-down for CDR1as and STAT3 respectively, with ~24% and ~11% leakage.

We also generated two stable lines carrying the light inducible Cre/Lox CasRx system, with or without a CDR1as targeting guide (PS2 from Fig. S2j), to test the knock-down properties of this system. Upon doxycycline and blue light stimulation, we observed on average ca. 50% knock-down of CDR1as, with ca. 25% leakage with doxycycline treatment and no leakage without (Fig. S2l).

In summary, we found that CasRx-mediated knock-downs could be induced upon photo-stimulation in HEK cells, with a tradeoff between activity and background. For example, the CRISPRa system combined with our CaSP1 promoter was very effective upon light stimulation, but it also had high background. CaSP2 had lower efficiency, but also lower leakage. This is common for inducible systems, and one has to adjust the experimental conditions to reach a predetermined goal. In this case, one can titrate the minimum amount of CasRx required for efficient knock-down of a given target, and then adjust the experimental setup to have the lit state exceeding that threshold. Further improvements may be possible, and similar approaches have been recently described (e.g. Blomeier et al., 2021). On the other hand, recent studies suggested that certain Cas13-mediated knock-downs can be prone to nonspecific off-target effects (Ai et al., 2022, Shi et al., 2021), which we did not observe in our experimental conditions but should be kept in mind when designing experimental setups and readouts.

We show in the main manuscript that the SCPTS and PA-Cre/Lox systems can be used to activate gene expression in organoids derived from hiPSC lines stably expressing the systems components, however while we have shown their potential and limitations when coupled with CasRx in HEK cells, it remains to be established how this works in organoids.

Importantly, the constructs and photo-stimulation approaches described here can be easily adjusted to control the expression of other CRISPR/Cas, RNAi or alternative RNA knock-down effectors. Finally, editing of endogenous gene by introducing LoxP cassettes in relevant locations may be used with the PA-Cre/Lox system to induce conditional knock-outs.

Attempts at Cas13 protein engineering for optogenetic RNA targeting.

We also attempted at engineering Cas13 proteins themselves for making their targeting activity light responsive, rather than their synthesis.

We first optimized a luciferase assay for assessing the targeting efficiency of Cas13 in human cells and designed several guides for PspCas13b (Cox et al., 2017), all eliciting high knock-down levels (Fig. S3a). We also designed a catalytically inactive version of PspCas13b (dCas13b) fused with the RNA silencing domain (RSD) of known RNA decay factors such as GW182 (TNRC6A and C, involved in microRNA-mediated targeting) and the endonuclease SMG6, which have been previously used for tethering experiments (Chen et al., 2009, Nicholson et al., 2014). Of these constructs, only the GW182 fusions were able to induce 50-60% luciferase knock-down, and only when targeting the 3'UTR (Fig. S3b-c; with three replicates, only TNRC6C had a significant effect).

Another Cas13 effector, CasRx, induced strong luciferase knock-down, while its catalytically inactive form did not (Fig. S3d).

To produce a light-inducible Cas13 system, we designed ten split PspCas13b pairs, which we fused with two distinct photo-dimer pairs (CRY/CIB and pMan/nMag) that should mediate the reconstitution of the full-length protein upon light stimulation (Kennedy et al., 2010, Kawano et al., 2015, Fig. S3e). For CasRx, we designed 14 split pairs and fused them with one pair of photo-dimers. We also split dCas13b as well as dCasRx and the GW182 RSDs and fused them in different arrangements with the photo-dimers (Fig. S3e).

We transfected all the split constructs together with the luciferase reporter and guide RNA and tested the targeting efficiency upon blue light stimulation for 24 hours (Fig. S3f). While light stimulation per se did not affect *wt* Cas13 knock-down efficiency (Fig. S3g), most of the Cas13b split pairs retained strong activity but reacted to light stimulation in an unexpected way, with the target being efficiently knocked down in the dark state, and the effect being reverted to different extents in the lit state (particularly, the 763 split position, Fig. S3h. Although we do not have an exhaustive explanation for this observation, we hypothesize that the split protein is still able to correctly fold in the dark state, while the change of conformation induced by photo-dimerization constrains target recognition, enzymatic activity and/or protein stability in the lit state, as observed in a different context in Zetsche et al., 2015. For the pMag/nMag PspCas13b-763 pair, we validated the initial screening result with additional independent replicates and confirmed that the light switch can induce up to 10-

fold knock-down of the luciferase reporter. We note however that the system is leaky, with approximately 50% knock-down happening in the lit (here to be considered the "inactive") state. The RSD photo-tethering approach, as well as the CasRx split approach, did not yield any candidate worth of further characterization (Fig. S3i-j).

In addition to the photodimerization/photothetering approaches, we also devised a few single-chain designs and a few split designs combined with "inverted" photodimer modules such as pdDronpa1 and LOVTRAP, which should dissociate in response to blue or cyan light stimulation (Zhou et al., 2017 and Wang et al., 2016). We fused two pdDronpa1 modules to Cas13b N- and C-termini, in a way that dimerization would cause steric hindrance at the interface with the guide:target RNA. This approach was leaky and did not produce significant light-dependent effects (Fig. S3k). We tried to use the functional Cas13b-763 split pair in combination with the LOVTRAP photodimer pair, but we still had strong target knock-down in the dark state (Fig. S3k). Finally, we inserted a LOV2-Ja module in a supposedly flexible region of CasRx (Zhang et al., 2018) and in the Cas13b-763 split site, reasoning that the rigid structure of the protein module would impair RNA targeting in the dark state, and its relaxation upon blue light stimulation would allow the protein to fold and acquire an active state. Again, we observed a strong knock-down in the dark state for Cas13b, and no significant activity of CasRx (Fig. S3k).

All these photo-stimulation experiments were performed with a LED board which can accommodate a 96-well plate within a cell culture incubator. Each of the 96 LEDs can be programmed independently and illuminate one single well with the desired ON/OFF pattern over time. We report the details for reproducing such device in the methods section and in a public repository (https://github.com/BIMSBbioinfo/casled).

From these experiments, we conclude that: I) Cas13b can be efficiently split to produce two protein modules which will naturally assemble and reconstitute the full-length functional protein; II) Cas13b can be fused with RNA silencing domains from GW182/TNRC6 proteins and be programmed to repress target genes by binding their 3'UTR; III) light-inducible target repression of engineered Cas13 proteins cannot be achieved with most of our designs, and when achieved it follows different principles and kinetics than expected. We note that points I) and II) may be of interest for synthetic biology applications. We found several functional split sites for Psp-Cas13b, which could be used for example for conditional knock-downs where the two protein modules are expressed from different promoters (as in Kempton et

al., 2020), or for fitting the Cas13b cassette into a compact viral vector (as in Chew et al., 2020). Also, we found that we can achieve target interference using a mutated Psp-Cas13b fused with domains of GW182 proteins, the effectors of microRNA silencing. Interestingly, the extent of silencing (ca. 50%) was consistent with that of microRNAs, and it worked only when tethering occurred on the 3'UTR, the natural platform for microRNA binding. These constructs might be used to manipulate gene expression or to study GW182 silencing activity.

Supplementary note 2: primers and gRNA sequences

Primers for *SHH* ISH probes.

SHH ISH fw: CCAAGGCACATATCCACTGC

T7 SHH ISH rv: GGATCCTAATACGACTCACTATAGGACCCGGTTGATGAGAATGGT

qRT-PCR primer pairs.

For qRT-PCR measurements of target RNAs, we used the following forward and reverse primers.

ASCL1 fw: CTTCACCAACTGGTTCTGAGG
ASCL1 rv: CAACGCCACTGACAAGAAAGC
CDR1as fw: ACGTCTCCAGTGTGCTGA
CDR1as rv: CTTGACACAGGTGCCATC
STAT3 fw: AACATGGAAGAATCCAACAACGG
STAT3 rv: TCTCAAAGGTGATCAGGTGCAG
GAPDH fw: AAGGTGAAGGTCGGAGTCAAC
GAPDH rv: GGGGTCATTGATGGCAACAATA
HPRT fw: ACCCCACGAAGTGTTGGATA
HPRT rv: AAGCAGATGGCCACAGAACT
SHH fw: AAGGATGAAGAAAACACCGGAGCG
SHH rv: ATATGTGCCTTGGACTCGTAGTAC
BMP4 fw: GCTGCTGAGGTTAAAGAGGAAACGA
BMP4 rv: CACTCGGTCTTGAGTATCCTGAG
FOXA2 fw: CCGTTCTCCATCAACAACCT
FOXA2 rv: GGGGTAGTGCATCACCTGTT
FOXG1 fw: CACTGCCTCCTAGCTTGTCC

FOXG1 rv: TGAACTCGTAGATGCCGTTG
OLIG2 fw: CCAGAGCCCGATGACCTTTTT
OLIG2 rv: CACTGCCTCCTAGCTTGTCC
NKX2-2 fw: CCGGGCCGAGAAAGGTATG
NKX2-2 rv: GTTTGCCGTCCCTGACCAA
NKX6-2 fw: GAGGACGACGACGAATACAAC
NKX6-2 rv: GTTCGAGGGTTTGTGCTTCTT

guide RNA sequences.

For most luciferase knock-downs, we used the previously validated (Abudayyeh et al., 2016) PS18 crRNA and non-targeting control (NT) for both Psp-Cas13b and CasRx, while the complementary sequence was cloned downstream of the Renilla luciferase reporter cassette in a psiCHECK-2 plasmid (Promega). The same target sequence was also cloned downstream of a TagRFP reporter cassette in an ePB-BSD-TT piggyback vector (see plasmids) for testing constitutive and light-inducible CasRx knock-downs. For the RSD tethering experiments, the 3'UTR was further swapped with another validated protospacer sequence[7], targeting the KRAS mRNA. The CDR1as crRNAs were designed on the CDR1as backsplice junction. The STAT3 mRNA crRNA sequence was taken from[8]. All guide RNA sequences were cloned into the pr026 plasmid, carrying either the Psp-Cas13b or CasRx direct repeat with two adjacent BbsI restriction sites for guide cloning (see plasmids).

NT guide: GTAATGCCTGGCTTGTCGACGCATAGTCTG
PS18 guide (luciferase and TagRFP 3'UTR): CATGCCTGCAGGTCGAGTAGATTGCTGT
KRAS guide (luciferase 3'UTR): AAACTATAATGGTGAATATCTTCAAATGATTT
CDR1as PS1 guide: GTGCCATCGGAAACCCTGGATATTGCAGAC
CDR1as PS2 guide: CCATCGGAAACCCTGGATATTGCAGACAC
STAT3 guide: ATCACAATTGGCTCGGCCCCCATTCCCACA

For the light-inducible CRISPRa experiments, we used the Tet6 sgRNA spacer sequence reported below, targeting CaSP1/2 and GAL4/UAS promoters. A sgRNA plasmid without any spacer cloned was used as a non-targeting guide control. We report below also all the tested guide RNA sequences for SHH and BMP4, designed after the Calabrese library (Sanson et al., 2018). All guides were cloned into the psgRNA2.0 plasmid carrying the SpCas9 sgRNA scaffold with two MS2 aptamers[3].

Non-targeting guide: GAACGACTAGTTAGGCGTGTA

ASCL1 guide: GCAGCCGCTCGCTGCAGCAG

Tet6 guide: GTCTTCGGAGGACAGTACTC

SHH guide 1: CATCAGAAGACAAGCTTGTG

SHH guide 2: AAAAAACGTAGTCTTCTTCA

SHH guide 3: TTTCCTAAGATAAAGGTGGG

BMP4 guide 1: CTCGCTCGCCTCCCTTTCTG

BMP4 guide 2: GGGGCTCCCATCCCCAGAAA

BMP4 guide 3: GCCTGCTAGGCGAGGTCGGG

Supplementary material-only references

A light-inducible CRISPR-Cas9 system for control of endogenous gene activation.
Polstein LR, Gersbach CA. Nat Chem Biol. 2015 Mar;11(3):198-200. doi: 10.1038/nchembio.1753. Epub 2015 Feb 9.

Photoactivatable CRISPR-Cas9 for optogenetic genome editing.
Nihongaki Y, Kawano F, Nakajima T, Sato M. Nat Biotechnol. 2015 Jul;33(7):755-60. doi: 10.1038/nbt.3245. Epub 2015 Jun 15. *In the text: Nihongaki et al., 2015b.*

Rapid blue-light-mediated induction of protein interactions in living cells.
Kennedy MJ, Hughes RM, Peteya LA, Schwartz JW, Ehlers MD, Tucker CL. Nat Methods. 2010 Dec;7(12):973-5. doi: 10.1038/nmeth.1524. Epub 2010 Oct 31.

A LOV2 domain-based optogenetic tool to control protein degradation and cellular function.
Renicke C, Schuster D, Usherenko S, Essen LO, Taxis C. Chem Biol. 2013 Apr 18;20(4):619-26. doi: 10.1016/j.chembiol.2013.03.005.

Engineered pairs of distinct photoswitches for optogenetic control of cellular proteins.
Kawano F, Suzuki H, Furuya A, Sato M. Nat Commun. 2015 Feb 24;6:6256. doi: 10.1038/ncomms7256.

A Single-Chain Photoswitchable CRISPR-Cas9 Architecture for Light-Inducible Gene Editing and Transcription.
Zhou XX, Zou X, Chung HK, Gao Y, Liu Y, Qi LS, Lin MZ. ACS Chem Biol. 2018 Feb 16;13(2):443-448. doi: 10.1021/acschembio.7b00603. Epub 2017 Sep 29.

Light-dependent RNA interference with nucleobase-caged siRNAs.

Mikat V, Heckel A. RNA. 2007 Dec;13(12):2341-7. doi: 10.1261/rna.753407. Epub 2007 Oct 19.

Optoribogenetic control of regulatory RNA molecules.
Pilsl S, Morgan C, Choukeife M, Möglich A, Mayer G. Nat Commun. 2020 Sep 24;11(1):4825. doi: 10.1038/s41467-020-18673-5.

RNA-guided cell targeting with CRISPR/RfxCas13d collateral activity in human cells
Shi P, Murphy MR, Aparicio AO, Kesner JS, Fang Z, Chen Z, Trehan A, Wu X. bioRxiv 2021.11.30.470032; doi: https://doi.org/10.1101/2021.11.30.470032

CRISPR/Cas13 effectors have differing extents of off-target effects that limit their utility in eukaryotic cells.
Ai Y, Liang D, Wilusz JE. Nucleic Acids Res 2022 Jun 24;50(11):e65. doi: 10.1093/nar/gkac159..

Blue Light-Operated CRISPR/Cas13b-Mediated mRNA Knockdown (Lockdown).
Blomeier T, Fischbach P, Koch LA, Andres J, Miñambres M, Beyer HM, Zurbriggen MD. Adv Biol (Weinh). 2021 May;5(5):e2000307. doi: 10.1002/adbi.202000307. Epub 2021 Feb 11.

Ago-TNRC6 triggers microRNA-mediated decay by promoting two deadenylation steps.
Chen CY, Zheng D, Xia Z, Shyu AB. Nat Struct Mol Biol. 2009 Nov;16(11):1160-6. doi: 10.1038/nsmb.1709. Epub 2009 Oct 18.

A novel phosphorylation-independent interaction between SMG6 and UPF1 is essential for human NMD.
Nicholson P, Josi C, Kurosawa H, Yamashita A, Mühlemann O. Nucleic Acids Res. 2014 Aug;42(14):9217-35. doi: 10.1093/nar/gku645. Epub 2014 Jul 22.
A split-Cas9 architecture for inducible genome editing and transcription modulation.
Zetsche B, Volz SE, Zhang F. Nat Biotechnol. 2015 Feb;33(2):139-42. doi: 10.1038/nbt.3149.

LOVTRAP: an optogenetic system for photoinduced protein dissociation.
Wang H, Vilela M, Winkler A, Tarnawski M, Schlichting I, Yumerefendi H, Kuhlman B, Liu R, Danuser G, Hahn KM. Nat Methods. 2016 Sep;13(9):755-8. doi: 10.1038/nmeth.3926. Epub 2016 Jul 18.

Structural Basis for the RNA-Guided Ribonuclease Activity of CRISPR-Cas13d.
Zhang C, Konermann S, Brideau NJ, Lotfy P, Wu X, Novick SJ, Strutzenberg T, Griffin PR, Hsu PD, Lyumkis D. Cell. 2018 Sep 20;175(1):212-223.e17. doi: 10.1016/j.cell.2018.09.001.

Multiple Input Sensing and Signal Integration Using a Split Cas12a System.
Kempton HR, Goudy LE, Love KS , Qi LS. Mol Cell. 2020 Apr 2;78(1):184-191.e3. doi: 10.1016/j.molcel.2020.01.016. Epub 2020 Feb 5.

A multifunctional AAV-CRISPR-Cas9 and its host response.
Chew WL, Tabebordbar M, Cheng JKW, Mali P, Wu EY , Ng AHM, Zhu K , Wagers AJ, Church GM. Nat Methods. 2016 Oct;13(10):868-74. doi: 10.1038/nmeth.3993. Epub 2016 Sep 5.

C2c2 is a single-component programmable RNA-guided RNA-targeting CRISPR effector.
Abudayyeh OO, Gootenberg JS, Konermann S, Joung J, Slaymaker IM, Cox DB, Shmakov S, Makarova KS, Semenova E, Minakhin L, Severinov K, Regev A, Lander ES, Koonin EV, Zhang F. Science. 2016 Aug 5;353(6299):aaf5573. doi: 10.1126/science.aaf5573. Epub 2016 Jun 2.

Optimized libraries for CRISPR-Cas9 genetic screens with multiple modalities.
Sanson KR, Hanna RE, Hegde M, Donovan KF, Strand C, Sullender ME, Vaimberg EW, Goodale A, Root DE, Piccioni F, Doench JG. Nat Commun. 2018 Dec 21;9(1):5416. doi: 10.1038/s41467-018-07901-8.

Figure S1

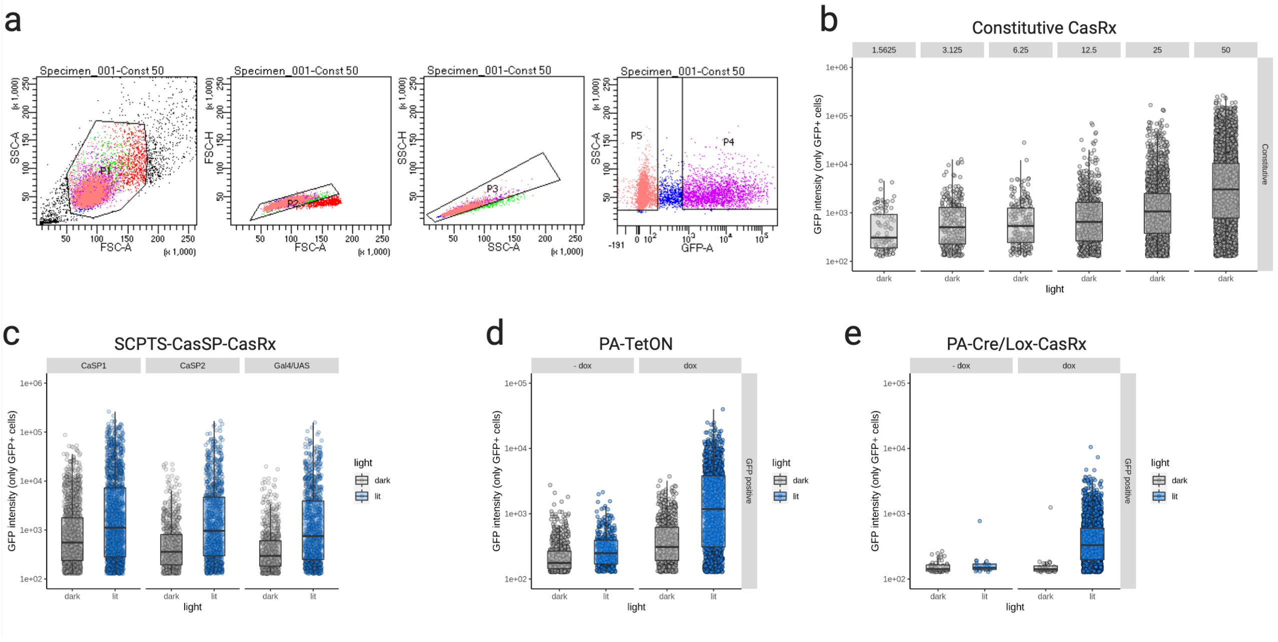

Supplementary Figure 1. Imaging validation by flow cytometry.

a. Representative (n=11 conditions tested in one replicate; here, the Constitutive 50 ng sample is shown) images of the gating strategy used for the FACS measurements of a GFP+ population in panels e-h. **b-e.** FACS validation (n=1) of GFP imaging data in HEK293T cells transfected with a titration of a constitutive (b) CasRx plasmid (ng plasmid are indicated on the top of each panel), the SCPTS CaSP1/2 and Gal4/UAS CasRx system (c), and stable lines with the PA-TetON (d) and the Cre/Lox (e) system with or without doxycycline. Cells were gated into GFP-negative and positive to better compare GFP levels in transfected cells, which are shown here. 10,000 total events were measured per condition. P-values (Wilcoxon-Mann-Whitney test) between dark and lit conditions are shown. All boxplots: center and bounds represent median, 25% and 75% quantiles. Whiskers represent 1.5× interquartile ranges.

Figure S2

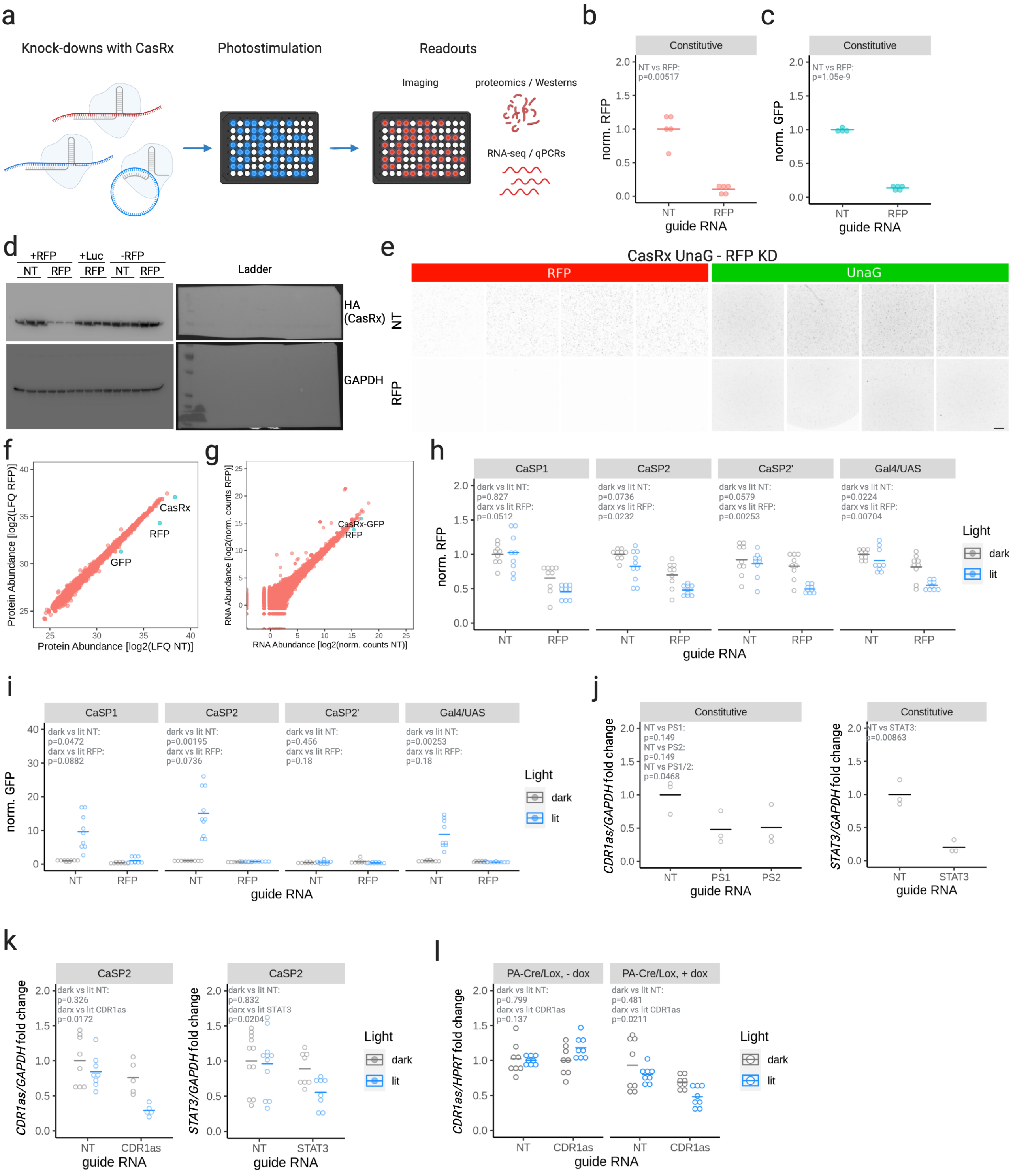

Supplementary Figure 2. Light-inducible RNA knock downs.

a. Reporter and endogenous RNA knock-downs with CasRx. **b-c.** Background-subtracted RFP and GFP intensity, with constitutive CasRx, non-targeting guide (NT, set to 1), or RFP-targeting guide (RFP). Horizontal bars: mean of all replicates (n=5 for all, n=4 for GFP NT). **d.** Western blot of HEK cells transfected with constitutive CasRx, non-targeting (NT) or RFP-targeting guide (RFP), +/- RFP or Luciferase reporter (+Luc), stained for HA tag (CasRx) and GAPDH (n=3). Right: ladder (PagerulerPlus). Blots were cut at 100 kDa before blocking. **e.** RFP and UnaG live fluorescence of HEK cells with constitutive UnaG-CasRx, RFP reporter, and

non-targeting (NT) or RFP-targeting guide (RFP) (n=4). Scale bar: 500 μm. **f.** Protein abundance (mean of n=3 biological, 2 technical shotgun proteomics replicates) in cells transfected with constitutive CasRx, RFP reporter, non-targeting guide (NT) or RFP-targeting guide (RFP). LFQ: log2 Label-Free Quantification intensity. Green: proteins with statistically significant and > two-fold change. **g.** RNA abundance (normalized counts from Deseq2, mean of n=2 biological replicates) in cells transfected with constitutive CasRx, RFP reporter, non-targeting guide (NT) or RFP-targeting guide (RFP). Green: transcripts with statistically significant difference between the two conditions, excluding four transcripts with high homology to the 18S rRNA (ribodepletion artifacts). **h-i.** As in b and c, for the promoters used with SCPTS (CaSP2' is CaSP2 with no GFP tag). Horizontal bars: mean of all replicates (n=9 for all, 10 for CaSP2 NT lit). **j.** qRT-PCR for *CDR1as* and *STAT3*, normalized on *GAPDH* and the non-targeting guide control, with constitutive CasRx and either a non-targeting guide (NT), *CDR1as*-targeting guides (PS1 and 2), or a *STAT3*-targeting guide. **k.** Same as j, for cells treated with the CaSP2 CasRx system in lit vs dark. Each dot represents a biological replicate (n=8, 5, 11, 8 for *CDR1as* with NT and CDR1as guides and for *STAT3* with NT and STAT3 guides respectively), horizontal bars represent mean of all replicates. *CDR1as* guide is PS2. **l.** As in j, in a stable Cre/Lox CasRx line, in dark vs lit, +/- doxycycline. *CDR1as* guide is PS2. Each dot represents a biological replicate (n=8), horizontal bars represent mean of all replicates. All p-values: Benjamini-Hochberg corrected two-sided t tests between dark and lit.

Figure S3

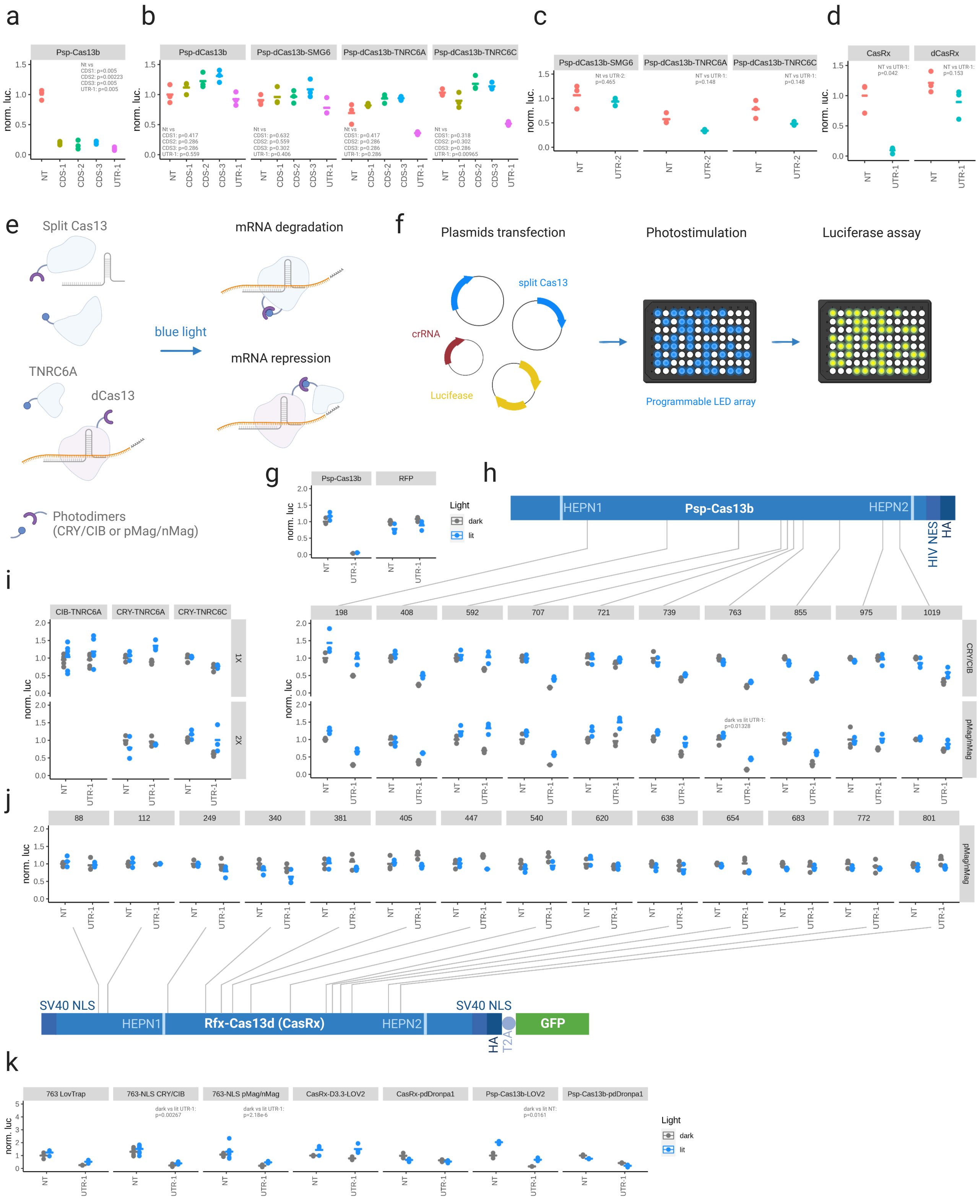

Supplementary Figure 3. Attempts at engineering a light-inducible Cas13 protein.

a. Normalized luciferase activity (Renilla/Firefly, non-targeting guide as reference) from HEK293T cells transfected with Psp-Cas13b, luciferase reporter, non-targeting guide (NT) or Renilla targeting guides in the coding sequence (CDS1-3) or 3'UTR (UTR-1). **b.** As in a., but with catalytically inactive Psp-Cas13b as it is or

fused with an RNA-silencing domain. **c.** As in b., with a different 3’UTR and corresponding guide (UTR-2). **d.** As in a., with CasRx and catalytically inactive CasRx, either the non-targeting (NT) or luciferase guide (UTR-1). **e.** Light-inducible Cas13 designs. Top: a split Cas13 in combination with a pair of photodimers. Bottom: a dCas13 and an RNA-silencing domain (RSD) in combination with a pair of photodimers. **f.** Experimental setup for testing light-inducible Cas13 designs: transfected cells in a 96-well plate are stimulated on a LED array for 24h, and the same plate is used for luciferase activity measurement. **g.** Positive (*wt* Psp-Cas13b) and negative (RFP) controls of the light-inducible Cas13 screening experiment, with non-targeting (NT) or luciferase-targeting (UTR-1) guide. **h.** Psp-Cas13b protein sequence, with its catalytic sites (HEPN), nuclear export signal (HIV NES), C-terminal HA tag. The position of each split site is indicated and linked to corresponding luciferase data. Split Psp-Cas13b are fused with CRY2/CIBN photodimer pairs (top) or pMag/nMag (bottom). Non-targeting dark control is set to 1 as reference for each design. **i.** As in h, for the phototethering designs with either 1 (top) or 2 (bottom) CIBN domains fused to Psp-dCas13b, and a CRY2 domain fused to the RNA-silencing domain from TNRC6A/C. **j.** Same as i., with split CasRx and pMag/nMag photodimers. **k.** Luciferase assay results for additional light-inducible designs as described in the Supplementary note. All experiments were performed in triplicate (n=3). Applies to all: horizontal bars indicate the mean for all replicates (n=3 for all except top-left in panel l n=6), Benjamini-Hochberg corrected two-sided t test p-values between conditions are indicated (only those <0.05 for panels g-k).
