# Supplementary material for: Spatiotemporal, optogenetic control of gene expression in organoids
Source: Nat Methods. 2023 Sep 21;20(10):1544–52. doi: 10.1038/s41592-023-01986-w (PMC10555836; doi:10.1038/s41592-023-01986-w)
Supplement: Supplementary file 2 — Reporting Summary [file 41592_2023_1986_MOESM2_ESM.pdf]

nature portfolio

Corresponding author(s): Ivano Legnini and Nikolaus Rajewsky

Last updated by author(s): Jun 7, 2023

Reporting Summary

Nature Portfolio wishes to improve the reproducibility of the work that we publish. This form provides structure for consistency and transparency in reporting. For further information on Nature Portfolio policies, see our Editorial Policies and the Editorial Policy Checklist.

Statistics

For all statistical analyses, confirm that the following items are present in the figure legend, table legend, main text, or Methods section.

n/a	Confirmed	
☐	☒	The exact sample size (*n*) for each experimental group/condition, given as a discrete number and unit of measurement
☐	☒	A statement on whether measurements were taken from distinct samples or whether the same sample was measured repeatedly
☐	☒	The statistical test(s) used AND whether they are one- or two-sided<br>*Only common tests should be described solely by name; describe more complex techniques in the Methods section.*
☐	☒	A description of all covariates tested
☐	☒	A description of any assumptions or corrections, such as tests of normality and adjustment for multiple comparisons
☐	☒	A full description of the statistical parameters including central tendency (e.g. means) or other basic estimates (e.g. regression coefficient) AND variation (e.g. standard deviation) or associated estimates of uncertainty (e.g. confidence intervals)
☐	☒	For null hypothesis testing, the test statistic (e.g. *F*, *t*, *r*) with confidence intervals, effect sizes, degrees of freedom and *P* value noted<br>*Give P values as exact values whenever suitable.*
☒	☐	For Bayesian analysis, information on the choice of priors and Markov chain Monte Carlo settings
☒	☐	For hierarchical and complex designs, identification of the appropriate level for tests and full reporting of outcomes
☒	☐	Estimates of effect sizes (e.g. Cohen's *d*, Pearson's *r*), indicating how they were calculated

Our web collection on statistics for biologists contains articles on many of the points above.

Software and code

Policy information about availability of computer code

Data collection	Commercial software provided with laboratory equipment: DLP LightCrafter 4500 Control Software (v3.1.0, Texas Instruments); StepOne plus Real-Time PCR system (StepOne software v2.3) Leica TCS SP8 microscope (LAS X v3.5.7.23225); Vilber Fusion FX imager (Evolution-CaptEdge - Fusion FX Edge 18.09) and Tecan M200 infinite Pro plate reader (i-control v1.1); BD FACSAriaIII (FACSDiVa v8.0.2), BioRender.
Data analysis	Fiji ImageJ 1.52p (Linux 64) and relevant plugins indicated in the Methods section; Micro-Manager 2.0; Spaceranger 1.2.0 (10X Genomics); RStudio 4.0 / R 4.0.4 and relevant packages indicated in the Methods section; MaxQuant v1.6.10.43; additional software developed in the Rajewsky lab: Spacemake, NovoSpaRc, Casled (https://github.com/rajewsky-lab/spacemake, https://github.com/rajewsky-lab/novosparc and https://github.com/BIMSBbioinfo/casled). Additional custom code describing the analyses performed in this manuscript has been deposited on Zenodo (10.5281/zenodo.6143560).

For manuscripts utilizing custom algorithms or software that are central to the research but not yet described in published literature, software must be made available to editors and reviewers. We strongly encourage code deposition in a community repository (e.g. GitHub). See the Nature Portfolio guidelines for submitting code & software for further information.

Data

Policy information about availability of data

All manuscripts must include a data availability statement. This statement should provide the following information, where applicable:

- Accession codes, unique identifiers, or web links for publicly available datasets
- A description of any restrictions on data availability
- For clinical datasets or third party data, please ensure that the statement adheres to our policy

All raw and processed sequencing data have been deposited on GEO (GSE185022).
Molecular Cartography data have been deposited on Zenodo (10.5281/zenodo.6143560).

Human research participants

Policy information about studies involving human research participants and Sex and Gender in Research.

Reporting on sex and gender	NA
Population characteristics	NA
Recruitment	NA
Ethics oversight	NA

Note that full information on the approval of the study protocol must also be provided in the manuscript.

Field-specific reporting

Please select the one below that is the best fit for your research. If you are not sure, read the appropriate sections before making your selection.

☒ Life sciences ☐ Behavioural & social sciences ☐ Ecological, evolutionary & environmental sciences

For a reference copy of the document with all sections, see nature.com/documents/nr-reporting-summary-flat.pdf

Life sciences study design

All studies must disclose on these points even when the disclosure is negative.

Sample size	Sample size was not predetermined. The number of biological replicates or the number of times a given experiment was performed was defined according to the standards in the field, depending on the experimental variability and readout.
Data exclusions	No data were excluded.
Replication	All findings were replicated (with few exceptions e.g. for validation of replicated findings with different techniques) and all replicates are shown either in the figures or in the associated raw data.
Randomization	No randomization was applied as the study did not involve large cohorts for treatment and control groups, but rather experimental replication and the use of different cell lines.
Blinding	Most eperiments were not performed blindly, as they were performed by one experimentalist at a time. Blinding was performed for the live imaging quantification shown in Fig. 1 and Ext. Data Fig. 1 as indicated in the Methods section.

Reporting for specific materials, systems and methods

We require information from authors about some types of materials, experimental systems and methods used in many studies. Here, indicate whether each material, system or method listed is relevant to your study. If you are not sure if a list item applies to your research, read the appropriate section before selecting a response.

Materials & experimental systems

n/a	Involved in the study
☐	☒ Antibodies
☐	☒ Eukaryotic cell lines
☒	☐ Palaeontology and archaeology
☒	☐ Animals and other organisms
☒	☐ Clinical data
☒	☐ Dual use research of concern

Methods

n/a	Involved in the study
☒	☐ ChIP-seq
☐	☒ Flow cytometry
☒	☐ MRI-based neuroimaging

Antibodies

Antibodies used	Primary antibodies: Anti-FOXA2 (R&D systems, #AF2400), Anti-OLIG2 (Sigma, #HPA003254-100UL), Anti-NKX6.1 (Sigma, #HPA036774-100UL), anti-Isl1/2 (DSHB, #39.4D5), anti-CHX10 (Novus Biologicals, #NBP1-85576), anti-GAPDH (Sigma #G8795-200UL) Anti-HA (NEB #3724S), anti-PDGFRB (Cell Signaling Technology, #3169), anti-mNeonGreen (ChromoTek #32F6), anti-DIG (Roche #11333089001). Secondary antobodies: Alexa Fluor 647 anti-Rabbit (Thermo Fisher, #A21244), Alexa Fluor 647 anti-Goat (Thermo Fisher, #A21447), HRP-conjugated anti-Rabbit (Dako #P0448), HRP-conjugated anti-mouse (Invitrogen #31430), .
Validation	Anti-FOXA2, OLIG2, NKX6.1, ISL1/2, CHX10 and mNeonGreen were used for immunofluorecence in control and SHH-induced organoids as described in the manuscript, with control organoids serving as negative control. For CHX10, the supplier reports also Western blot validation of HEK cells expressing the recombinant antigen. The anti-HA antibody for western blotting was previously validated by us analyzing samples of non-transfected versus transfected HEK cells with HA-labelled proteins. A similar validation is reported by the supplier in HeLa cells. All other antibodies are broadly used in the field and were not validated by us.

Eukaryotic cell lines

Policy information about cell lines and Sex and Gender in Research

Cell line source(s)	XM001 hiPSC cells were kindly provided by the Heiko Lickert lab under a Material Transfer Agreement with our institution. Gibco Human 18 Episomal iPS line 1E6, cat#A18944, lot #2036936. HEK293T cells were kindly provided by the Markus Landthaler lab at our institution.
Authentication	None of the cell lines were authenticated by us. XM001 and Gibco Human 18 are maintained and validated by the Stem Cells core facility and Organoid platform at our institution.
Mycoplasma contamination	All batches and lines were regularly tested for mycoplasma contamination by PCR and resulted negative.
Commonly misidentified lines (See ICLAC register)	HEK293T cells appear in the register of commonly misidentified cell lines.

Flow Cytometry

Plots

Confirm that:

☒ The axis labels state the marker and fluorochrome used (e.g. CD4-FITC).

☒ The axis scales are clearly visible. Include numbers along axes only for bottom left plot of group (a 'group' is an analysis of identical markers).

☒ All plots are contour plots with outliers or pseudocolor plots.

☒ A numerical value for number of cells or percentage (with statistics) is provided.

Methodology

Sample preparation	HEK293T cells were harvested with 0.05% tripsin, washed twice in PBS and resuspended in 1% BSA PBS prior to flow cytometry analysis.
Instrument	BD FACSAriaIII Cell Sorter Model No: 648282-31
Software	FACSDiVa v8.0.2
Cell population abundance	10,000 events were measured for each sample.
Gating strategy	Gating information is provided in the Supplementary materials. Briefly, GFP positive and negative populations were defined to restrict the analysis to surely transfected cells (GFP positive).

☒ Tick this box to confirm that a figure exemplifying the gating strategy is provided in the Supplementary Information.
